# Quality of Chronic Kidney Disease Management in Canadian Primary Care

**DOI:** 10.1001/jamanetworkopen.2019.10704

**Published:** 2019-09-04

**Authors:** Aminu K. Bello, Paul E. Ronksley, Navdeep Tangri, Julia Kurzawa, Mohamed A. Osman, Alexander Singer, Allan K. Grill, Dorothea Nitsch, John A. Queenan, James Wick, Cliff Lindeman, Boglarka Soos, Delphine S. Tuot, Soroush Shojai, K. Scott Brimble, Dee Mangin, Neil Drummond

**Affiliations:** 1 Division of Nephrology and Immunology, Department of Medicine, University of Alberta, Edmonton, Alberta, Canada; 2 Department of Community Health Sciences, University of Calgary, Calgary, Alberta, Canada; 3 Department of Medicine, Max Rady College of Medicine, Winnipeg, Manitoba, Canada; 4 Department of Family Medicine, University of Manitoba, Winnipeg, Manitoba, Canada; 5 Department of Family and Community Medicine, University of Toronto, Toronto, Ontario, Canada; 6 Department of Non-Communicable Disease Epidemiology, London School of Hygiene and Tropical Medicine, London, United Kingdom; 7 Canadian Primary Care Sentinel Surveillance Network, Department of Family Medicine, Queen’s University, Kingston, Ontario, Canada; 8 Department of Medicine, University of Calgary, Calgary, Alberta, Canada; 9 Department of Family Medicine, University of Alberta, Edmonton, Alberta, Canada; 10 Department of Family Medicine, University of Calgary, Calgary, Alberta, Canada; 11 Division of Nephrology, University of California, San Francisco; 12 Kidney Health Research Institute, University of California, San Francisco; 13 Division of Nephrology, Department of Medicine, McMaster University, Hamilton, Ontario, Canada; 14 Department of Family Medicine, McMaster University, Hamilton, Ontario, Canada

## Abstract

**Importance:**

Although patients with chronic kidney disease (CKD) are routinely managed in primary care settings, no nationally representative study has assessed the quality of care received by these patients in Canada.

**Objective:**

To evaluate the current state of CKD management in Canadian primary care practices to identify care gaps to guide development and implementation of national quality improvement initiatives.

**Design, Setting, and Participants:**

This cross-sectional study leveraged Canadian Primary Care Sentinel Surveillance Network data from January 1, 2010, to December 31, 2015, to develop a cohort of 46 162 patients with CKD managed in primary care practices. Data analysis was performed from August 8, 2018, to July 31, 2019.

**Main Outcomes and Measures:**

The study examined the proportion of patients with CKD who met a set of 12 quality indicators in 6 domains: (1) detection and recognition of CKD, (2) testing and monitoring of kidney function, (3) use of recommended medications, (4) monitoring after initiation of angiotensin-converting enzyme inhibitors (ACEIs) or angiotensin II receptor blockers (ARBs), (5) management of blood pressure, and (6) monitoring for glycemic control in those with diabetes and CKD. The study also analyzed associations of divergence from these quality indicators.

**Results:**

The cohort comprised 46 162 patients (mean [SD] age, 69.2 [14.0] years; 25 855 [56.0%] female) with stage 3 to 5 CKD. Only 4 of 12 quality indicators were met by 75% or more of the study cohort. These indicators were receipt of an outpatient serum creatinine test within 18 months after confirmation of CKD, receipt of blood pressure measurement at any time during follow-up, achieving a target blood pressure of 140/90 mm Hg or lower, and receiving a hemoglobin A_1c_ test for monitoring diabetes during follow-up. Indicators in the domains of detection and recognition of CKD, testing and monitoring of kidney function (specifically, urine albumin to creatinine ratio testing), use of recommended medications, and appropriate monitoring after initiation of treatment with ACEIs or ARBs were not met. Only 6529 patients (18.4%) with CKD received a urine albumin test within 6 months of CKD diagnosis, and 3954 (39.4%) had a second measurement within 6 months of an abnormal baseline urine albumin level. Older age (≥85 years) and CKD stage 5 were significantly associated with not satisfying the criteria for the quality indicators across all domains. Across age categories, younger patients (aged 18-49 years) and older patients (≥75 years) were less likely to be tested for albuminuria (314 of 1689 patients aged 18-49 years [18.5%], 1983 of 11 919 patients aged 75-84 years [61.6%], and 614 of 5237 patients aged ≥85 years [11.7%] received the urine albumin to creatinine ratio test within 6 months of initial estimated glomerular filtration rate <60 mL/min per 1.73 m^2^; *P* < .001). Patients aged 18 to 49 years were less commonly prescribed recommended medications (222 of 2881 [7.7%]), whereas patients aged 75 to 84 years were prescribed ACEIs or ARBs most frequently (2328 of 5262 [44.2%]; *P* < .001).

**Conclusions and Relevance:**

The findings suggest that management of CKD across primary care practices in Canada varies according to quality indicator. This study revealed potential priority areas for quality improvement initiatives in Canadian primary care practices.

## Key Points

**Question:**

What is the current status of chronic kidney disease management in Canadian primary care practice settings?

**Findings:**

In this cross-sectional study of 46 162 individuals with moderate to severe chronic kidney disease who received care in primary care practices in Canada, 4 of 12 quality indicators were met by 75% or more of the study cohort. Guideline-recommended care relating to monitoring and testing for albuminuria and recommended medication use were identified as gaps in management of chronic kidney disease.

**Meaning:**

The findings suggest that although most patients received high-quality care, there are gaps in treatment that may be key priority areas for quality improvement.

## Teaser

This cross-sectional study assesses chronic kidney disease management in Canadian primary care practices to identify care gaps to guide development and implementation of national quality improvement initiatives.

## Introduction

Chronic kidney disease (CKD) is a frequently treated condition at health care systems, both globally and within Canada, with a prevalence of approximately 10% in the general population.^1,2,3^ Most patients with CKD are at low risk of progression to end-stage kidney disease (ESKD) and are ideally managed in primary care settings.^4^ Organizations such as Kidney Disease: Improving Global Outcomes, the UK National Institute for Health and Clinical Excellence,^5^ and the Canadian Society of Nephrology^6^ provide recommendations regarding the management of patients with CKD to reduce the risk of adverse consequences of ESKD and cardiovascular disease. Despite these guidelines, variability in care continues.^7,8,9^

Regular quality audits at local, provincial or state, and national levels could identify variations in care and inform resource allocation, primary care physician training, education, and other quality improvement activities.^10,11^ Furthermore, assessment benchmarks for quality are the first step to evaluating innovations aimed at creating high-functioning and sustainable health systems.^12^ A few studies^7,9,13^ have examined quality of care for patients with CKD in primary care settings using provincial (regional) data. To our knowledge, no studies have examined pan-Canadian performance in meeting quality-of-care indicators for CKD management in primary care, as has been done in other settings.^14,15,16,17^

National chronic disease surveillance systems, such as the Canadian Primary Care Sentinel Surveillance Network (CPCSSN), have been designed to facilitate national quality improvement studies to improve chronic disease management.^15,16,17,18^ Understanding data from Canadian primary care may indicate gaps in care processes and demonstrate a proof of concept for the use of CPCSSN data to inform targeted priorities for improvement in management of patients with chronic diseases. The key objectives of this study were to define the current state of CKD management in Canadian primary care practices based on existing guidelines and to stratify key results by population demographics.

## Methods

### Design and Participants

**Figure 1.**
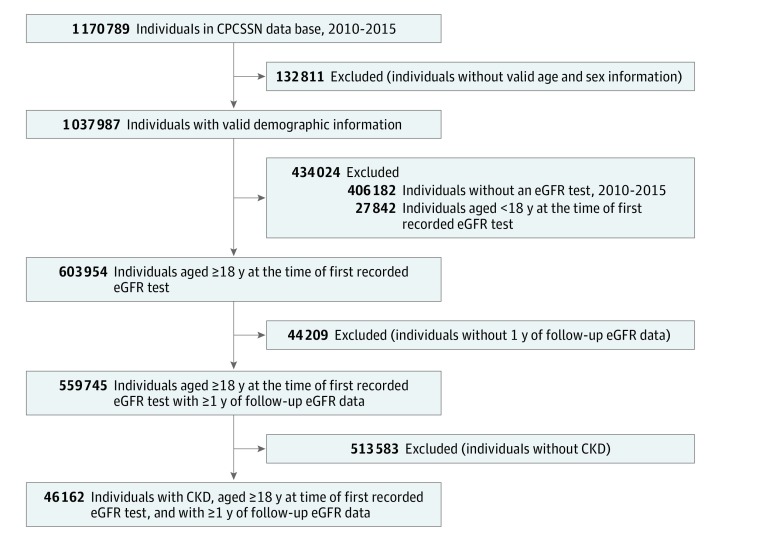
Development of the Chronic Kidney Disease (CKD) Cohort The steps of developing a cohort of patients with CKD who were managed in primary care between January 1, 2010, and December 31, 2015, from Canadian Primary Care Sentinel Surveillance Network (CPCSSN) data repository are shown. Chronic kidney disease was defined as at least 2 estimated glomerular filtration rate (eGFR) measurements less than 60 mL/min per 1.73 m^2^ at least 90 days apart.

**Figure 2.**
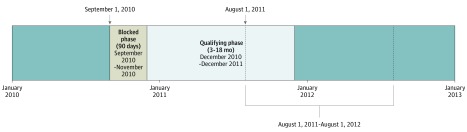
Decision Process for Identification of Individuals With Chronic Kidney Disease (CKD) A sample timeline of the decision process followed to identify patients with CKD (defined as having ≥2 estimated glomerular filtration rate [eGFR] measurements <60 mL/min per 1.73 m^2^ within at least 3 months but not more than 18 months) in the Canadian Primary Care Sentinel Surveillance Network data repository. Qualifying eGFR indicates an eGFR value less than 60 mL/min per 1.73 m^2^; blocked phase, period of 90 days after the first qualifying eGFR measurement at which no second eGFR measurement was considered confirmatory of CKD; qualifying phase, period of 3 to 18 months after the first qualifying eGFR measurement that a second eGFR measurement confirms CKD and qualifies the patient to be included in the study; and follow-up period, 1 year after confirmation of CKD to assess the use of appropriate medications.

This cross-sectional study used a national database (CPCSSN data) to develop a cohort of patients with CKD managed in primary care from January 1, 2010, to December 31, 2015 (Figure 1). Data analysis was performed from August 8, 2018, to July 31, 2019. We examined prevalent CKD (defined based on expert guideline criteria)^19^ during the study period and determined quality indicators for CKD care in patients who met the case criteria. Patients were identified as having CKD if they had at least 2 estimated glomerular filtration rate (eGFR) measurements less than 60 mL/min/1.73 m^2^ within a period of at least 3 months but not more than 18 months (Figure 2). Only those with moderate-to-advanced CKD (stages 3-5) were eligible. Patients with ESKD undergoing dialysis or who had received a kidney transplant were excluded. We followed the Strengthening the Reporting of Observational Studies in Epidemiology (STROBE) reporting guideline to outline the findings. This study was approved by the CPCSSN Surveillance and Research Standing Committee and the University of Alberta Health Research Ethics Committee. The CPCSSN has received a waiver of the requirement to obtain individual patient consent to include their deidentified data in its data set unless they have specifically opted out. As data custodians, sentinels permit this use of the data on behalf of their patients.

### Setting and Data Sources

The CPCSSN is composed of 13 regional networks that form a national disease surveillance system that collects primary care data from 9 of the 13 provinces and territories in Canada.^20^ Data in the CPCSSN repository are derived from primary care electronic medical records (EMRs) and are cleaned, coded, deidentified, and made available to users for research, surveillance, evaluation, and quality improvement purposes.^21^

### Definition and Derivation of Quality Indicators of CKD Management

Quality indicators for CKD care in primary care practices were derived from the published expert guideline and those developed by the Canadian experts. The Canadian Society of Nephrology published a guideline for the management of CKD 2 years before the onset of this study in 2008.^19^

**Figure 3.**
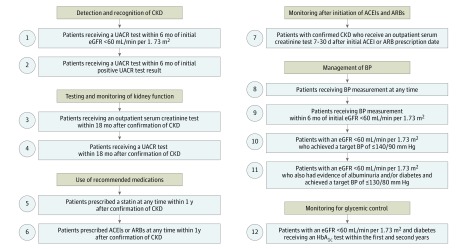
Overview of Quality of Care Indicators Studied The 12 quality indicators for patients with chronic kidney disease (CKD) in primary care used in the study are shown. The 12 indicators were categories under the domains of detection and recognition of CKD, testing and monitoring of kidney function, use of recommended medications, monitoring after initiation of treatment with angiotensin-converting enzyme inhibitors (ACEIs) and angiotensin II receptor blockers (ARBs), management of blood pressure, and monitoring for glycemic control. BP indicates blood pressure; eGFR, estimated glomerular filtration rate; HbA_1c_, glycated hemoglobin; and UACR, urine albumin to creatinine ratio.

We examined and adapted 12 quality indicators based on previously published data and guidelines.^7,19^ The indicators were categorized under the domains of detection and recognition of CKD, testing and monitoring of kidney function, use of recommended medications, monitoring after initiation of treatment with angiotensin-converting enzyme inhibitors (ACEIs) and angiotensin II receptor blockers (ARBs), management of blood pressure, and monitoring for glycemic control (Figure 3). The cutoff for being considered as having achieved each quality-of-care indicator was 75% of patients who reached the target during the study period.

### Covariates

To understand the variability in quality indicators, we divided the cohort of patients with CKD into 4 categories for subgroup analysis using validated CPCSSN case definitions: patients without hypertension or diabetes, patients with hypertension only, patients with diabetes only, and patients with hypertension and diabetes.^22^ For further analysis, we stratified quality indicator data by CKD stage (stages 3-5), age category, and sex. All variables were extracted from patient EMRs available in the CPCSSN data repository.

### Statistical Analysis

Patient demographic and clinical characteristics were tabulated descriptively using proportions or means as appropriate. The proportion of patients who met the CKD management criteria for the quality indicators were calculated for the overall cohort and by comorbid subgroup, with χ^2^ tests of the differences between subgroups indicating statistically significant differences in quality of care. We used binomial regression models to identify key demographic characteristics associated with not receiving specified care. We reported the estimated risk ratio (RR) and corresponding 95% CI for each association for the variables assessed and presented these graphically using forest plots. Missing data were handled by listwise deletion because the missingness was assumed to be random. All analyses were performed using Stata, version 14.2 (StataCorp).^23^ A 1-sided *P* < .05 was considered to be statistically significant.

## Results

### Baseline Demographics

The baseline cohort was composed of 46 162 patients (mean [SD] age, 69.2 [14.0] years; 25 855 [56.0%] female) from primary care practices in Canada identified with stages 3 to 5 CKD during the study period. A total of 24 368 patients (68.6%) had stage 3A CKD, with a mean eGFR value of 47.7 mL/min per 1.73 m^2^. A total of 38 545 patients (83.5%) had at least 1 comorbid condition. A total of 7603 patients (16.5%) had neither diabetes nor hypertension, 6770 (14.7%) had diabetes without hypertension, 16 930 (36.7%) had hypertension only, and 14 859 (32.2%) had both diabetes and hypertension (Table 1).

**Table 1.** Cohort Characteristics Overall and by Comorbid Status^a^

Characteristic	Overall (N = 46 162)	Patients With CKD				*P* Value
		Without Diabetes or Hypertension (n = 7603 [16.5%])	With Diabetes (n = 6770 [14.7%])	With Hypertension (n = 16 930 [36.7%])	With Diabetes and Hypertension (n = 14 859 [32.2%])	
Age group, y						
18-49	4535 (9.8)	1017 (13.4)	1471 (21.7)	763 (4.5)	1284 (8.6)	<.001
50-64	11 137 (24.1)	1718 (22.6)	2172 (32.1)	2836 (16.8)	4411 (29.7)	
65-74	12 286 (26.6)	1785 (23.5)	1546 (22.8)	4501 (26.6)	4454 (30.0)	
75-84	12 840 (27.8)	1971 (25.9)	1220 (18.0)	5990 (35.4)	3659 (24.6)	
≥85	5364 (11.6)	1112 (14.6)	361 (5.3)	2840 (16.8)	1051 (7.1)	
Age, mean (SD), y	69.2 (14.0)	68.6 (16.1)	62.2 (15.5)	73.5 (12.1)	67.8 (12.4)	
Female sex	25 855 (56.0)	4659 (61.3)	3071 (45.4)	10 700 (63.2)	7425 (49.9)	<.001
CKD stage at first qualifying eGFR measurement^b^						
3A	24 368/35 517 (68.6)	5270/6951 (75.8)	2300/3484 (66.0)	10 749/15 666 (68.6)	6049/9416 (64.2)	<.001
3B	845/35 5177 (23.8)	1296/6951 (18.6)	841/3484 (24.1)	3846/15 666 (24.6)	2474/9416 (26.3)	
4	2290/35 517 (6.5)	304/6951 (4.4)	266/3484 (7.6)	942/15 666 (6.0)	778/9416 (8.3)	
5	402/35 517 (1.1)	81/6951 (1.2)	77/3484 (2.2)	129/15 666 (0.8)	115/9416 (1.2)	
eGFR, mean (SD), mL/min per 1.73 m^2^	47.7 (10.5)	49.4 (10.0)	46.8/3484 (11.4)	47.8 (10.1)	46.6 (10.9)	NA

Abbreviations: CKD, chronic kidney disease; eGFR, estimated glomerular filtration rate; NA, not applicable.

^a^ Data are presented as number or number/total number (percentage) of patients unless otherwise indicated.

^b^ First qualifying eGFR measurement is the first measurement of 60 mL/min per 1.73 m^2^ or less.

### Overview of Quality of CKD Management

Only 4 quality indicators were successfully met in 75% or more of the cohort (Table 2 and eFigure 1 in the Supplement). These indicators were receipt of an outpatient serum creatinine test within 18 months after confirmation of CKD, receipt of blood pressure measurement at any time during follow-up, achieving a target blood pressure of 140/90 mm Hg or lower, and receiving a hemoglobin A_1c_ test for monitoring diabetes during follow-up. These indicators were in the domains of testing and monitoring of kidney function (eGFR), management of blood pressure, and glycemic control. Criteria for the remaining indicators (in the domains of detection and recognition of CKD [monitoring of albuminuria], use of recommended medications, and appropriate monitoring after initiation of treatment with ACEIs or ARBs) were not met in at least 75% of the cohort.

**Table 2.** Quality-of-Care Indicators for CKD, Blood Pressure, and Glycemic Control Overall and by Comorbid Status

Domain and Quality Indicator	No. (%) of Patients					*P* Value
	Overall	CKD				
		Without Diabetes or Hypertension	With Diabetes	With Hypertension	With Diabetes and Hypertension	
Detection and recognition of CKD						
Patients receiving UACR test within 6 mo of initial eGFR <60 mL/min per 1.73 m^2^	6529 (18.4)	472 (6.8)	1129 (32.4)	1607 (10.3)	3321 (35.3)	<.001
Patients receiving UACR test within 6 mo of initial positive UACR test result	3954 (39.4)	254 (43.9)	1130 (38.4)	413 (35.5)	2157 (40.4)	.001
Testing and monitoring of kidney function						
Patients with an outpatient SCr test in the 18 mo after the confirmation of CKD	27 221 (85.5)	4552 (77.3)	2913 (89.2)	11 668 (84.5)	8088 (91.2)	<.001
Patients with a UACR test in the 18 mo following the confirmation of CKD	8599 (27.0)	581 (9.9)	1485 (45.5)	2219 (16.1)	4314 (48.7)	<.001
Use of recommended medications						
Patients prescribed a statin any time in the 1 y after the confirmation of CKD	11 672 (36.7)	1198 (20.3)	1399 (42.9)	4613 (33.4)	4462 (50.3)	<.001
Patients prescribed an ACEI or ARB any time in the 1 y after the confirmation of CKD who have evidence of proteinuria and/or diabetes	6964 (30.5)	57 (27.3)	1278 (18.9)	551 (54.9)	5078 (30.5)	<.001
Monitoring after initiation of treatment with ACEIs or ARBs						
Patients with confirmed CKD who receive an outpatient SCr test 7-30 d after initial ACEI or ARB prescription date	659 (26.7)	69 (26.3)	73 (27.8)	334 (28.5)	183 (23.8)	.14
Management of BP						
Patients receiving BP measurement at any time	34 941 (75.7)	4998 (65.7)	5120 (75.6)	13 064 (77.2)	11 759 (79.1)	<.001
Patients receiving BP measurement within 6 mo of initial eGFR <60 mL/min per 1.73 m^2^	13 914 (30.1)	1904 (25.0)	1263 (18.7)	6332 (37.4)	4415 (29.7)	<.001
Patients with eGFR <60 mL/min per 1.73 m^2^ achieving a target BP of ≤140/90 mm Hg	15 467 (81.4)	2574 (89.2)	1730 (86.8)	6582 (78.9)	4581 (79.3)	<.001
Patients with eGFR <60 mL/min per 1.73 m^2^ achieving a target BP of ≤130/80 mm Hg, who have evidence of proteinuria and/or diabetes	4689 (59.6)	81 (71.1)	1249 (67.8)	339 (53.2)	3020 (57.3)	<.001
Monitoring for glycemic control, patients with eGFR <60 mL/min per 1.73 m^2^ and diabetes who have HbA_1c_ tested within the first and second years						
0-1 y	11 073 (85.9)	NA	3018 (85.7)	NA	8055 (86.0)	.66
1-2 y	8626 (66.9)	NA	2266 (64.4)	NA	6360 (67.9)	<.001

Abbreviations: ACEI, angiotensin-converting enzyme inhibitor; ARB, angiotensin II receptor blocker; BP, blood pressure; CKD, chronic kidney disease; eGFR, estimated glomerular filtration rate; HbA_1c_, glycated hemoglobin; NA, not applicable; SCr, serum creatinine; UACR, urine albumin to creatinine ratio.

### Detection and Recognition of CKD and Monitoring of Kidney Function

Overall, 6529 patients with CKD (18.4%) received follow-up urine albumin to creatinine ratio (UACR) testing within 6 months of CKD diagnosis. In subgroup analysis, detection was significantly less common among patients without diabetes or hypertension (472 [6.8%]) and most common among patients with diabetes and hypertension (3321 [35.3%]) (*P* < .001) (Table 2). A total of 3954 patients (39.4%) had UACR confirmatory testing within 6 months after a positive albuminuria test result. A total of 27 221 patients (85.5%) received a follow-up serum creatinine and eGFR test in the 18 months after the confirmation of CKD. In subgroup analysis, there was a statistically significant difference in the number of patients receiving an outpatient serum creatinine test: 8088 patients (91.2%) with comorbid hypertension and diabetes vs 4552 patients (77.3%) without comorbid diabetes or hypertension (*P* < .001). Overall, 8599 patients (27.0%) received a follow-up UACR test in the 18 months after the confirmation of CKD. In subgroup analysis, there was a statistically significant difference in the number of patients receiving a follow-up UACR: 4314 patients (48.7%) with comorbid hypertension and diabetes vs 581 patients (9.9%) without comorbid diabetes or hypertension (*P* < .001) (Table 2).

### Use of Recommended Medications and Appropriate Monitoring After Initiation of Treatment With ACEIs or ARBs

A total of 11 672 patients (36.7%) were prescribed statins, and 6964 (30.5%) were prescribed ACEIs or ARBs within 1 year of confirmation of CKD. Overall, 5078 patients (30.5%) with evidence of albuminuria and/or diabetes were prescribed ACEIs or ARBs within 1 year of CKD diagnosis. Both ACEIs and ARBs were most commonly prescribed for patients with comorbid hypertension (551 [54.9%]) and least commonly prescribed for those with comorbid diabetes (1278 [18.9%]) (Table 2). Only 659 patients (26.7%) with confirmed CKD received an outpatient serum creatinine test 7 to 30 days after the initial ACEI or ARB prescription date (Table 2). Patients with comorbid diabetes and hypertension (183 [23.8%]) were tested least frequently, whereas those with hypertension only (334 [28.5%]) were tested most frequently.

### Management of Blood Pressure and Monitoring for Glycemic Control

A total of 34 941 patients (75.7%) had at least 1 blood pressure measurement during the study period. Subgroup analysis revealed that blood pressure had been measured for 4998 (65.7%) of those with CKD but without diabetes or hypertension and for 11 759 (79.1%) of those with diabetes and hypertension (Table 2). Only 13914 patients (30.1%) had their blood pressure measured within 6 months of a qualifying eGFR measurement. Variations among comorbid groups revealed that blood pressure was more commonly measured among those with hypertension (6332 [37.4%]) (Table 2). Overall, 15 467 patients (81.4%) met the guideline-concordant blood pressure target of 140/90 mm Hg or less (Table 2). A total of 4689 patients (59.6%) with albuminuria and/or diabetes met the guideline-concordant target blood pressure measure of 130/80 mm Hg or less (Table 2). Most patients with CKD and diabetes had a glycated hemoglobin test within the first (11 073 [85.9%]) and second (8626 [66.9%]) years of the study (Table 2).

### Variations Across Disease Stage, Comorbid Status, Age, and Sex

Across CKD stages, delivery of guideline-concordant care was more common with each progressive stage with the exception of stage 5 (eTable 1 in the Supplement). Older age (≥85 years) and CKD stage 5 were significantly associated with not satisfying the criteria for the quality indicators across all domains (eTable 1 and eTable 2 in the Supplement). Across age categories, younger patients (aged 18-49 years) and older patients (≥75 years) were less likely to be tested for albuminuria (314 of 1689 patients aged 18-49 years [18.5%], 1983 of 11 919 patients aged 75-84 years [61.6%], and 614 of 5237 patients aged ≥85 years [11.7%] received the UACR test within 6 months of initial eGFR <60 mL/min per 1.73 m^2^; *P* < .001) (eTable 2 in the Supplement). Patients aged 18 to 49 years were less commonly prescribed recommended medications (222 of 2881 [7.7%]), whereas patients aged 75 to 84 years were prescribed ACEIs or ARBs most frequently (2328 of 5262 [44.2%]) (*P* < .001) (eTable 2 in the Supplement). Stratification by sex revealed that for 5 of 7 indicators (detection and recognition of CKD [2 indicators], testing and monitoring of kidney function [2 indicators], and use of recommended medications [1 indicator]), care was more likely to conform to recommendations for men than for women (eTable 3 in the Supplement).

### Factors Associated With Lower Achievement of Quality Indicators

The factors associated with not receiving a UACR test within 6 months and 18 months of a qualifying eGFR are shown in eFigure 2 and eFigure 3 in the Supplement. Factors associated with not being prescribed a statin are given in eFigure 4 in the Supplement, for not being prescribed ACEIs or ARBs for patients who had evidence of proteinuria and/or diabetes in eFigure 5 in the Supplement, and for not receiving a blood pressure measurement and achieving targets in eFigures 6-9 in the Supplement.

### Variations Across Physician Characteristics

Overall, no association between age or sex (or a combination) of physician and adherence to guidelines was found (eTable 4 in the Supplement).

## Discussion

In this national study of more than 46 000 Canadian individuals with stage 3 to 5 CKD managed in primary care, we identified gaps in the quality of CKD care related to monitoring and testing for albuminuria as well as use of recommended medications to reduce risk of progression to ESRD and prevent cardiovascular events. To our knowledge, this is the first study to examine quality indicators for CKD management in Canadian primary care at a national level and to study associations of variance.

Overall, results of this study suggest a need to understand underlying reasons for and appropriateness of variance in particular quality indicators. Among the 12 indicators examined, only 4 revealed satisfactory performance, thus providing important opportunities to improve CKD management. The 4 quality indicators that revealed satisfactory performance were in the domains of testing and monitoring of kidney function (ie, eGFR measurements after confirmation of CKD), management of blood pressure, and monitoring for glycemic control. The domains for improvement in the quality of care were concerned with the detection and recognition of CKD risk associated with cardiovascular disease (ie, albuminuria measurements), use of recommended medications, and monitoring of kidney function after prescription of ACEIs and ARBs.

We found that the presence of stage 5 CKD and older ages were associated with a lower likelihood of meeting the quality indicators. There are several possible reasons why these gaps may exist. First, these findings may relate to clinical decisions that reflect increased patient complexity or a more supportive approach to care. Multimorbidity is common in older adults,^24^ meaning that single-disease approaches to guiding treatment may be inappropriate and potentially harmful if treatment targets are applied indiscriminately while ignoring medical complexity. For example, it may be appropriate to not prescribe medications for a specific individual given their multimorbidity even though it may seem appropriate based on guidelines.^25,26^ The reasons are complex and include drug-drug and drug-disease interactions, a patient’s treatment priorities, the overall burden of medical care, and limited lifespan benefit. Understanding the difference between appropriate variance and inappropriate (low-quality) care can inform future quality improvement initiatives. Evidence-based medicine is predicated on patient-centered decision-making, which is one of the core mechanisms by which primary care achieves better population outcomes. In this model, individuals are free to choose not to engage with recommended care, and it is valid for a patient to make a choice that is consistent with their values despite incongruence with guideline recommendations.

After stratification by stage of CKD and sex, with the general exception of stage 5, each progressive stage of CKD was associated with increased conformity to guideline-recommended care for 5 of 7 quality indicators in the domains of detection and recognition of CKD, testing and monitoring of kidney function, use of recommended medications, and monitoring of kidney function after an ACEI or ARB was prescribed. This finding might be related to early stages of CKD often remaining undiagnosed; more advanced stages of CKD are more readily identified and therefore better managed. Findings that reveal sex differences in disease management have been reported in different disciplines (eg, women receive less guideline-concordant care for myocardial infarction^27^ and heart failure^28^). Specific to CKD, the sex differences associated with heart failure described in the literature relate to disease epidemiology, prognosis, and progression.^29,30^ The novel finding of sex differences in quality of care for CKD is an area for future study. Possible implications for sex differences in disease management could include the development of new approaches to disease identification and modified training materials for medical practitioners.^11,30^

A major implication to practice and quality improvement initiatives is that the management of CKD varied across indicators. The worst-performing indicator and a finding of significant concern was that only 18.4% of patients received a follow-up UACR test within 6 months of CKD diagnosis. The association of older age with assessed variables provides direction for the development of quality improvement interventions. One possible explanation is that some health care professionals may not consider an eGFR measurement within the range of stage 3A CKD to indicate a risk of disease progression to ESKD in older people. They might consider such a measurement as reflecting the physiologic changes associated with aging because CKD seldom progresses in the absence of albuminuria. Thus, some experts have asked for an age-calibrated classification for CKD.^31^ The other quality indicator at variance with guideline concordance was follow-up albuminuria tests when indicated, which was not well met. Associations for not receiving this test included older age and rural residency, the latter reflecting previous findings that patients with CKD living in rural settings may receive lower quality of care than patients living in urban settings.^32^ The rural-urban health disparity has been the subject of many reports^33,34^ and initiatives in Canada, but work still needs to be done to address this issue. These findings may facilitate further research and investment into alternative modes of health care delivery to patients in rural communities, such as telemedicine and electronic consultation. However, the increased likelihood of detection in the subgroup with diabetes and hypertension suggests an increased understanding of risk factors for albuminuria in CKD.

Furthermore, the indicator for the domain of monitoring ACEI and ARB use (ie, outpatient serum creatinine testing 7-30 days after the initial prescription date) was not met and is an area for further work with the aim of understanding variation and appropriateness as a basis for quality improvement. Performance associated with this quality indicator improved as CKD stage progressed, which suggests greater awareness of the importance of monitoring medication use during advanced stages of the disease.

Results related to blood pressure quality indicators were mixed. Most patients had their blood pressure measured at least once during the study, whereas only a few patients had it measured within 6 months of CKD diagnosis. The quality indicator of patients who met the guideline-concordant blood pressure target of 140/90 mm Hg or less was met; however, there was room for improvement in the quality indicator of patients with proteinuria or diabetes meeting the guideline-concordant blood pressure target of 130/80 mm Hg or less. The variables significantly associated with not achieving a target blood pressure were older age, stage 3A CKD, and living in rural areas. Older age is associated with increased risk of comorbidities; therefore, elevated blood pressure is common. Treatment of hypertension with pharmacotherapy in older age is also associated with an increased risk of falls and serious injury, and treatment thresholds are therefore a balance of risks and benefits requiring a more individualized approach to care.^35^ The finding that those with stage 3A CKD were unlikely to achieve a target blood pressure remains unexplained. Our finding that living in rural areas was associated with nonconformance with target blood pressure levels is supported by results from a population-based study^9^ that found that patients with diabetes and CKD living in rural or remote parts of Alberta, Canada, were less likely to meet process-based outcomes (eg, glycated hemoglobin and albuminuria measurements, use of recommended medications) than were their urban counterparts. These findings underscore the importance of targeted intervention to address geographic disparities in CKD care. For instance, the use of electronic consultations (asynchronous electronic communication between physicians) was found to improve access to specialist advice in remote communities in Canada.^36^

In addition to the patient-related factors (demographic, clinical, and laboratory) outlined in our results, we specifically examined physician factors and found no association between age or sex (or a combination) of physician and adherence to guidelines. It is important to recognize the wider context of physician factors in implementation of guideline recommendations, which has implications in understanding concordance and variation.^37^ For example, the volume of guideline recommendations for primary care is increasing at a rate that is not sustainable for implementation. For a primary care physician, it would take 7 hours a day to follow all preventive recommendations and 10 hours a day to follow recommendations for 10 chronic diseases. These data are based on an assessment in the mid-2000s, and guideline proliferation continues.^37,38^ How should a primary care physician prioritize smoking cessation vs urine protein analysis? Primary care physicians may prioritize patients according to which factor seems likely to be most associated with patients’ outcomes and focus on quality improvement initiatives that are most important for population health improvement. This approach may improve implementation and adoption of guidelines in general.

This study builds on a previous study^39^ that used data from CPCSSN to estimate the prevalence of CKD being managed in primary care practices across Canada. This current work provides an in-depth assessment of the current practice pattern and variations in care for CKD to understand areas of appropriate and inappropriate variation within the context of multimorbidity, patient-centered care, and primary care service delivery to support quality improvement that is most meaningful for patients.

### Limitations

This study had several limitations. We limited our analysis to data from patients with moderate to advanced stages of CKD (stages 3-5) because patients with early stages of CKD cannot be readily identified based on eGFR measurements alone. As a result, we were not able to capture the quality of care received by patients with early-stage CKD, which is often asymptomatic. Another limitation of the study relates to the representativeness of the cohort to the general Canadian population. Our cohort tended to be older than the general Canadian population and included slightly more women than men than in the general population. Moreover, data in the CPCSSN repository are based on information available from the source EMRs; gaps in data quality (particularly related to completeness and capture) may have underestimated actual clinical performance associated with the indicators considered in this study, which has limited our ability to use other CKD markers, such as dipstick proteinuria. Furthermore, the nature of the data made it difficult to establish temporality with clinical situations that could limit the application of some of the quality indicators in practice, for example, the use of ACEIs or ARBs in the context of hyperkalemia and/or hypotension.

In addition, even though the Canadian Society of Nephrology guideline for the management of CKD was published 2 years before the onset of this study, differential uptake of its recommendations among primary care physicians could be fraught with inherent complexities. Physician-related factors (eg, age, sex, years in practice, time, and resources), patient factors, and practice environment contextual factors (academic vs community based, rural vs urban, and regulation) inform adoption of guidelines into practice.^40^ Moreover, it is also widely recognized that lack of awareness of the availability of the guideline and familiarity with its details were common barriers to implementation in patient care.^41^ These issues are relevant to the interpretation of our findings. Some primary care physicians in Canada might not have been aware of the existence of the CKD management guideline, and this awareness might have come to them over time in their practice outside the scope of the study. Clinical practice guidelines are often produced by specialty societies, as was the CKD management guideline, and it would be challenging for primary care physicians to keep up to date and adopt all of the guidelines into practice. These data may provide an opportunity to engage with relevant primary care organizations in Canada, such as the College of Family Physicians of Canada and other primary care professional societies, to close the identified gaps and facilitate uptake of the guideline for optimal kidney care.

## Conclusions

The findings suggest that management of CKD across primary care practices in Canada varies according to quality indicator. This study revealed potential priority areas for quality improvement initiatives in Canadian primary care practices.

## Notes

Supplement. **eFigure 1.** Performance related to quality indicators **eFigure 2.** Associations for not receiving a UACR test within 6 months following the confirmation of CKD **eFigure 3.** Associations for not receiving a UACR test within 18 months following the confirmation of CKD **eFigure 4.** Associations for not being prescribed a statin at any time in the 1 year following the confirmation of CKD **eFigure 5.** Associations for not being prescribed an ACE inhibitor or ARB at any time in the 1 year following the confirmation of CKD **eFigure 6.** Associations for not receiving a blood pressure measurement at any time **eFigure 7.** Associations for not receiving a blood pressure measurement within six months of initial eGFR measurement **eFigure 8.** Associations for not achieving a target BP of ≤140/90mmHg, among those with blood pressure measure after CKD diagnosis **eFigure 9.** Associations for not achieving a target BP of ≤130/80mmHg, among those with confirmed CKD and evidence of albuminuria and/or diabetes **eTable 1.** Quality of care indicators for CKD, overall and by comorbid status, and disease stage **eTable 2.** Quality of care indicators for CKD, overall and by comorbid status and age categories **eTable 3.** Quality of care indicators for CKD, overall and by comorbid status, and sex **eTable 4.** Variations of quality of care indicators for CKD, across physician characteristics (age and gender)
