# Supplementary material for: Quality of Chronic Kidney Disease Management in Canadian Primary Care
Source: JAMA Netw Open. 2019 Sep 4;2(9):e1910704. doi: 10.1001/jamanetworkopen.2019.10704 (PMC6727682; doi:10.1001/jamanetworkopen.2019.10704)
Supplement: Supplement. — eFigure 1. Performance related to quality indicators eFigure 2. Associations for not receiving a UACR test within 6 months following the confirmation of CKD eFigure 3. Associations for not receiving a UACR test within 18 months following the confirmation of CKD eFigure 4. Associations for not being prescribed a statin at any time in the 1 year following the confirmation of CKD eFigure 5. Associations for not being prescribed an ACE inhibitor or ARB at any time in the 1 year following the confirmation of CKD eFigure 6. Associations for not receiving a blood pressure measurement at any time eFigure 7. Associations for not receiving a blood pressure measurement within six months of initial eGFR measurement eFigure 8. Associations for not achieving a target BP of ≤140/90mmHg, among those with blood pressure measure after CKD diagnosis eFigure 9. Associations for not achieving a target BP of ≤130/80mmHg, among those with confirmed CKD and evidence of albuminuria and/or diabetes eTable 1. Quality of care indicators for CKD, overall and by comorbid status, and disease stage eTable 2. Quality of care indicators for CKD, overall and by comorbid status and age categories eTable 3. Quality of care indicators for CKD, overall and by comorbid status, and sex eTable 4. Variations of quality of care indicators for CKD, across physician characteristics (age and gender) [file jamanetwopen-2-e1910704-s001.pdf]

Supplementary Online Content

Bello AK, Ronksley PE, Tangri N, et al. Quality of chronic kidney disease management in Canadian primary care. *JAMA Netw Open*. 2019;2(9):e1910704. doi:10.1001/jamanetworkopen.2019.10704

eFigure 1. Performance related to quality indicators

eFigure 2. Associations for not receiving a UACR test within 6 months following the confirmation of CKD

eFigure 3. Associations for not receiving a UACR test within 18 months following the confirmation of CKD

eFigure 4. Associations for not being prescribed a statin at any time in the 1 year following the confirmation of CKD

eFigure 5. Associations for not being prescribed an ACE inhibitor or ARB at any time in the 1 year following the confirmation of CKD

eFigure 6. Associations for not receiving a blood pressure measurement at any time

eFigure 7. Associations for not receiving a blood pressure measurement within six months of initial eGFR measurement

eFigure 8. Associations for not achieving a target BP of ≤140/90mmHg, among those with blood pressure measure after CKD diagnosis

eFigure 9. Associations for not achieving a target BP of ≤130/80mmHg, among those with confirmed CKD and evidence of albuminuria and/or diabetes

eTable 1. Quality of care indicators for CKD, overall and by comorbid status, and disease stage

eTable 2. Quality of care indicators for CKD, overall and by comorbid status and age categories

eTable 3. Quality of care indicators for CKD, overall and by comorbid status, and sex

eTable 4. Variations of quality of care indicators for CKD, across physician characteristics (age and gender)

This supplementary material has been provided by the authors to give readers additional information about their work.

© 2019 Bello AK et al. *JAMA Network Open.*

eFigure 1. Performance related to quality indicators

Indicator	< 25%	25–50%	50–75%	> 75%
**Detection and recognition of CKD**				
1. Patients receiving a **UACR** test within 6 mos. of initial eGFR < 60 ml/min/1.73 m^2	■			
2. Patients receiving a **UACR** test within 6 mos. of initial positive UACR test		■		
**Testing and monitoring of kidney function**				
3. Patients receiving an outpatient **SCr** test within 18 mos. after confirmation of CKD				■
4. Patients receiving a **UACR** test within 18 mos. after confirmation of CKD		■		
**Use of recommended medications**				
5. Patients prescribed a **statin** within 1 year after confirmation of CKD		■		
6. Patients with evidence of proteinuria and/or diabetes prescribed **ACEIs** or **ARBs** within 1 year after confirmation of CKD		■		
**Monitoring after initiation of ACEIs/ARBs**				
7. Patients with confirmed CKD receiving an outpatient **SCr** test 7 to 30 days after initial ACEIs/ARBs prescription date		■		
**Management of blood pressure**				
8. Patients receiving **BP** measurement at any time				■
9. Patients receiving **BP** measurement within 6 mos. of initial eGFR < 60 ml/min/1.73 m^2		■		
10. Patients with eGFR < 60 ml/min/1.73 m^2 achieving a **target BP of ≤ 140/90mmHg**				■
11. Patients with evidence of proteinuria and/or diabetes and eGFR < 60 ml/min/1.73 m^2 achieving a **target BP of ≤ 130/80mmHg**			■	
**Monitoring for glycemic control**				
12. Patients with GFR < 60 ml/min/1.73 m^2 and diabetes receiving **HbA1c** test within the 1st and 2nd years				■

■ > 75% of patients met the quality indicator; ■ 50–75% of patients met the quality indicator; ■ 25–50% of patients met the quality indicator; ■ < 25% of patients met the quality indicator.
UACR = urine albumin-to-creatinine ratio; eGFR = estimated glomerular filtration rate; SCr = serum creatinine; ACEIs = angiotensin-converting enzyme inhibitors; ARBs = angiotensin-receptor blockers; BP = blood pressure; HbA1c = glycated hemoglobin test.

© 2019 Bello AK et al. *JAMA Network Open.*

eFigure 2. Associations for not receiving a UACR test within 6 months following the confirmation of CKD

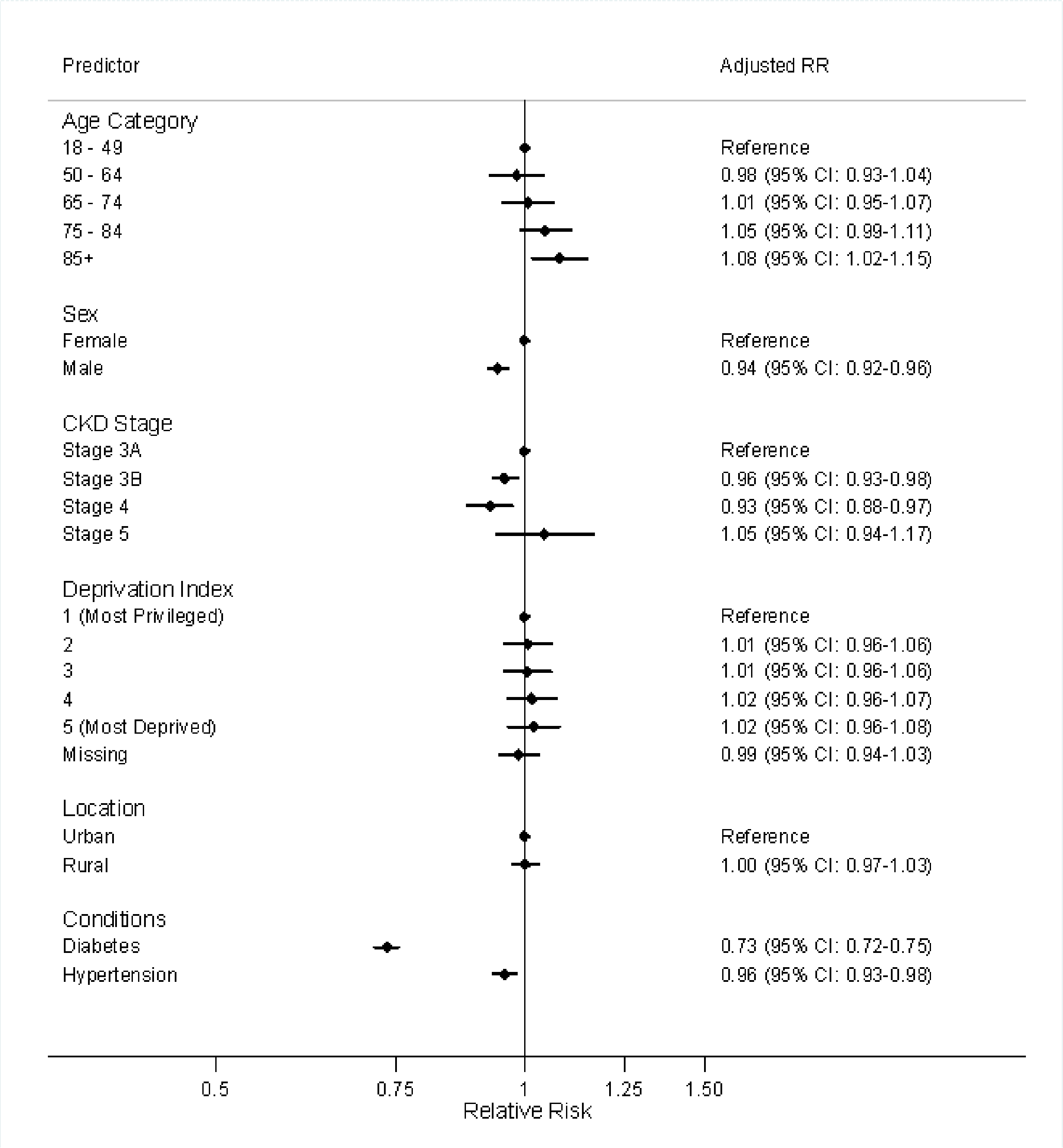

CKD=chronic kidney disease; CI= confidence interval; RR= risk ratio; UACR=urine albumin-to-creatinine ratio

© 2019 Bello AK et al. *JAMA Network Open.*

eFigure 3. Associations for not receiving a UACR test within 18 months following the confirmation of CKD

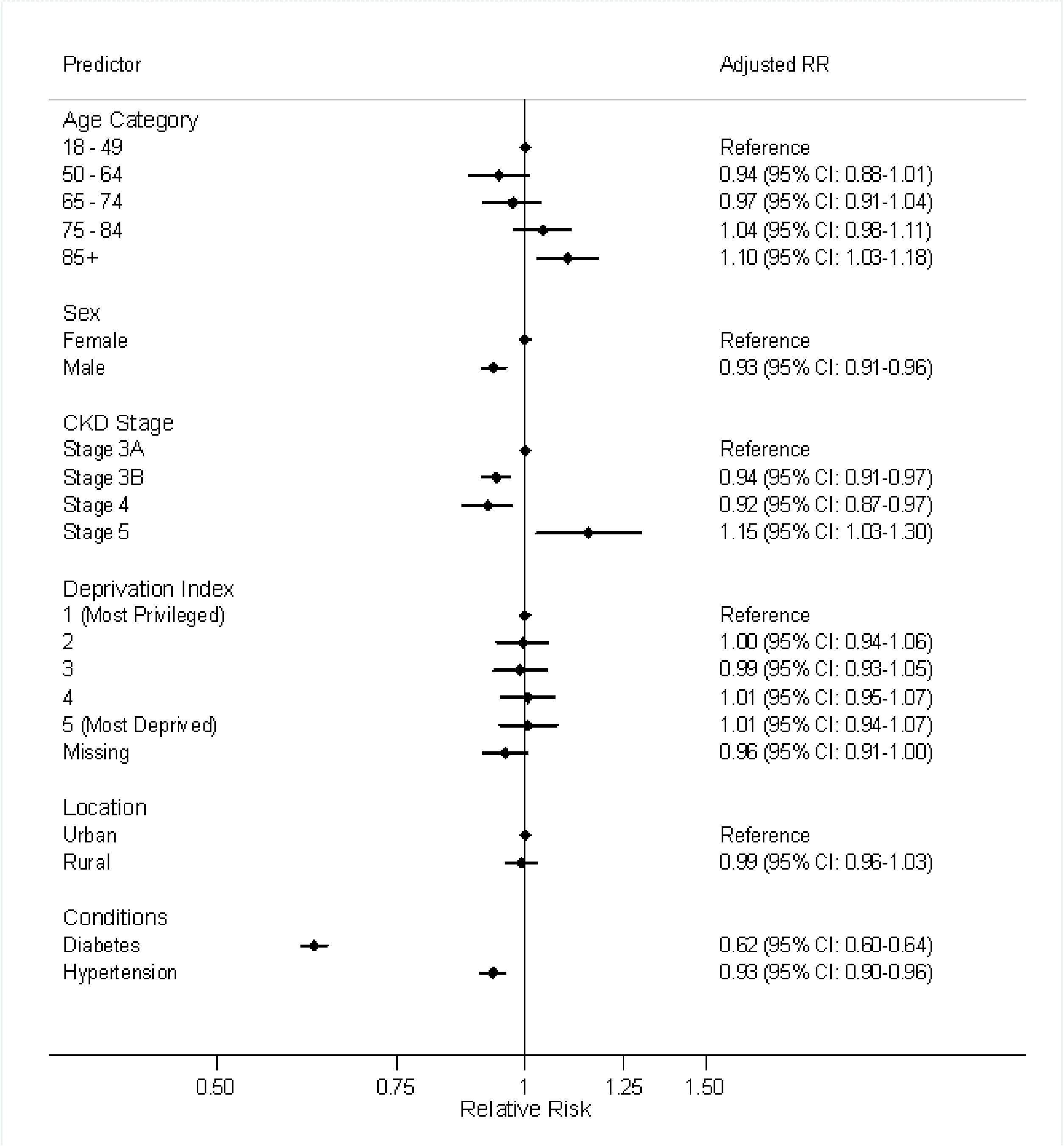

CKD=chronic kidney disease; CI= confidence interval; RR= risk ratio; UACR=urine albumin-to-creatinine ratio

© 2019 Bello AK et al. *JAMA Network Open.*

eFigure 4. Associations for not being prescribed a Statin at any time in the 1 year following the confirmation of CKD

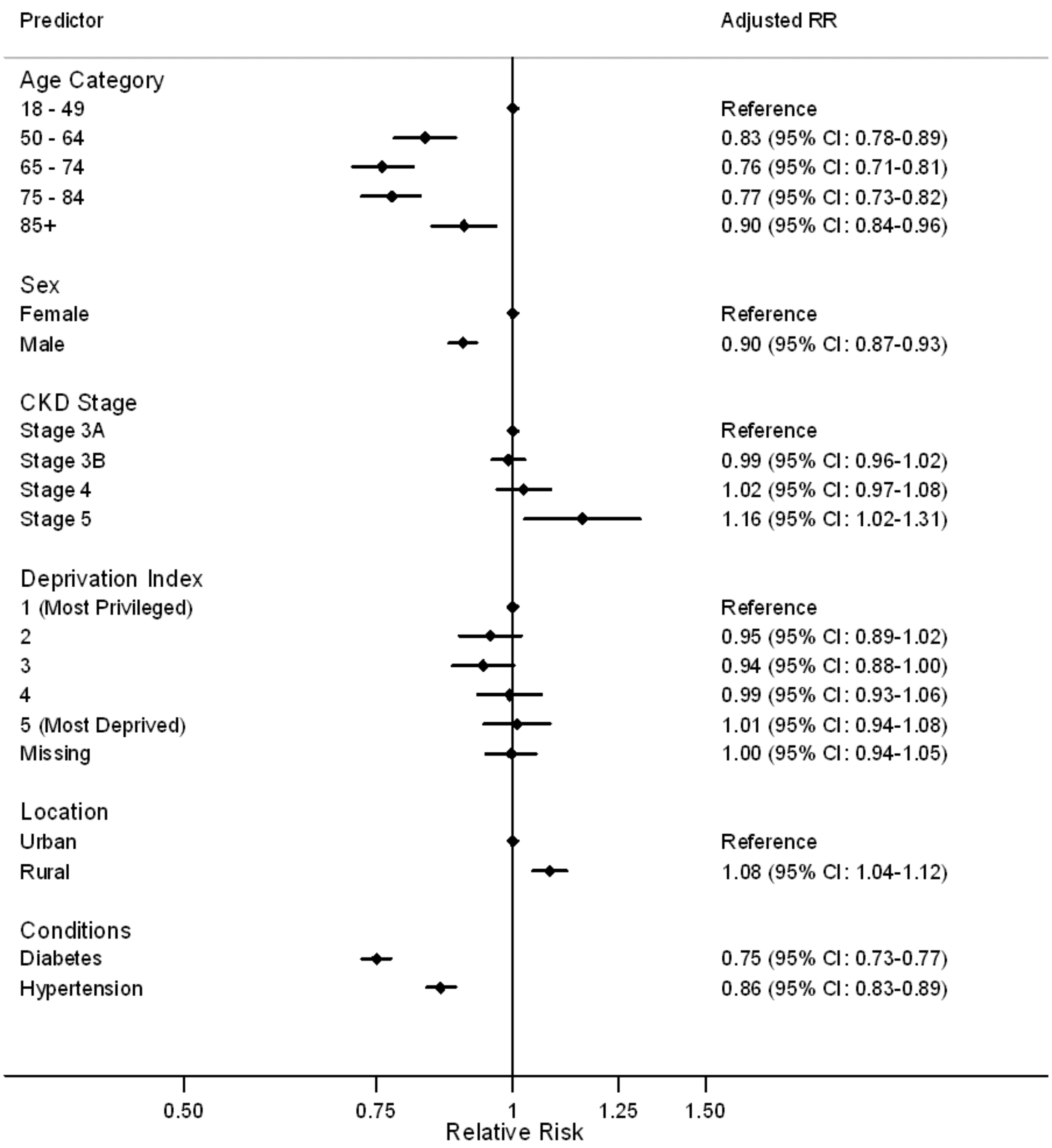

CKD=chronic kidney disease; CI= confidence interval; RR= risk ratio

© 2019 Bello AK et al. *JAMA Network Open.*

eFigure 5. Associations for not being prescribed an ACE inhibitor or ARBs at any time in the 1 year following the confirmation of CKD

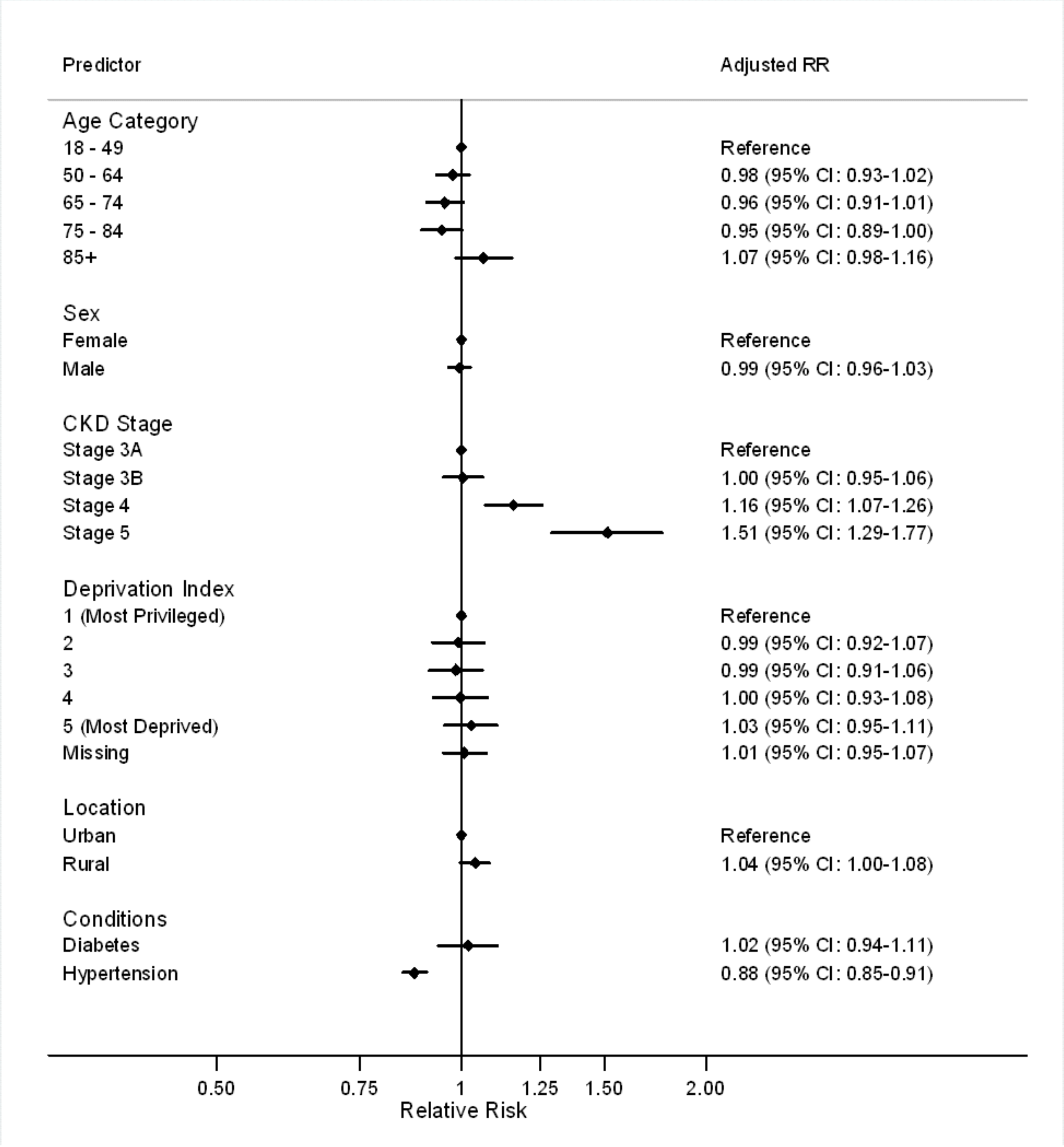

ACEI=angiotensin-converting enzyme inhibitors; ARBs=angiotensin-receptor blockers; CKD=chronic kidney disease; CI= confidence interval; RR= risk ratio

© 2019 Bello AK et al. *JAMA Network Open.*

eFigure 6. Associations for not receiving a blood pressure measurement at any time

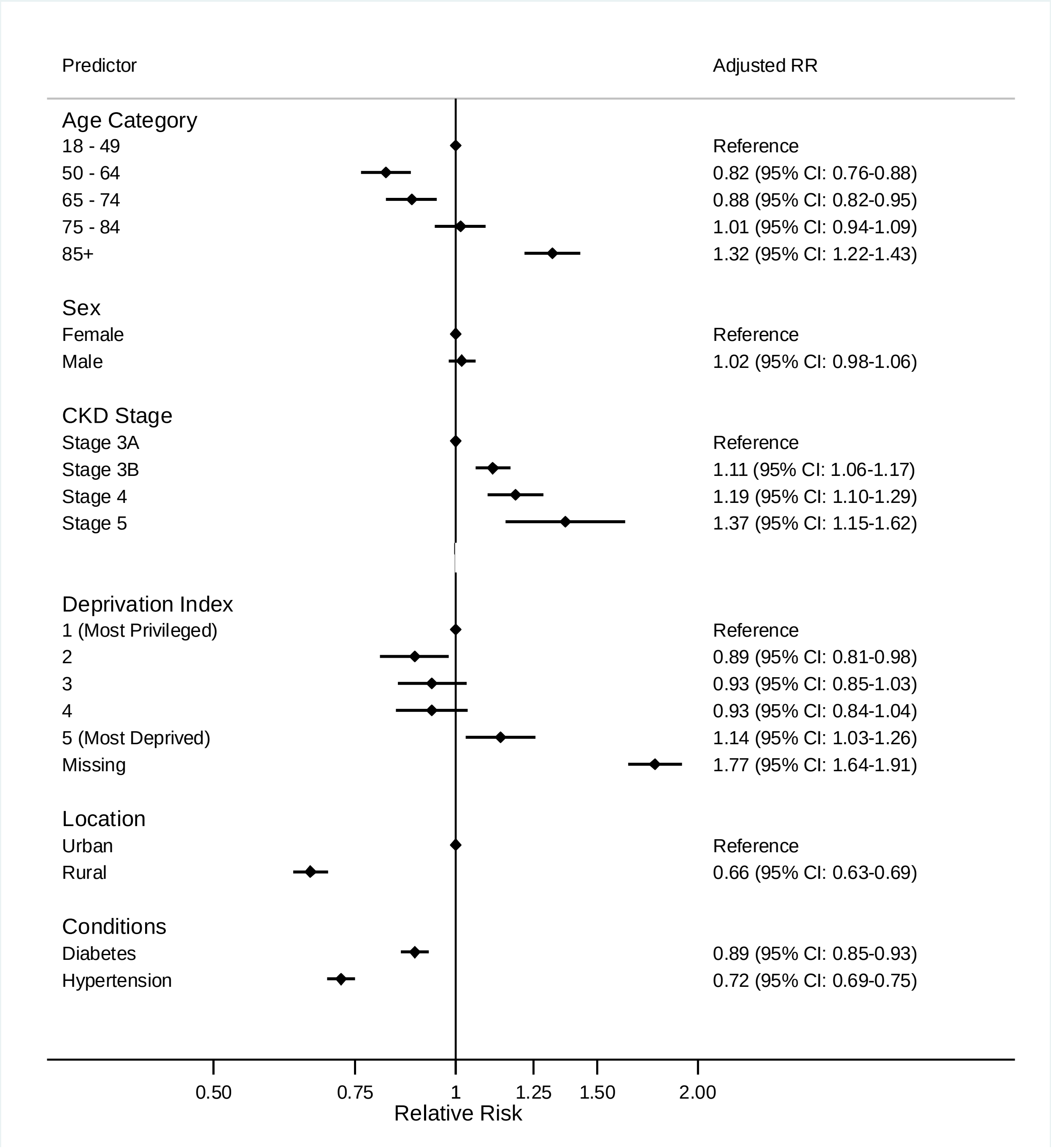

CKD=chronic kidney disease; CI= confidence interval; RR= risk ratio

© 2019 Bello AK et al. *JAMA Network Open.*

eFigure 7: Associations for not receiving a blood pressure measurement within six months of initial eGFR measurement

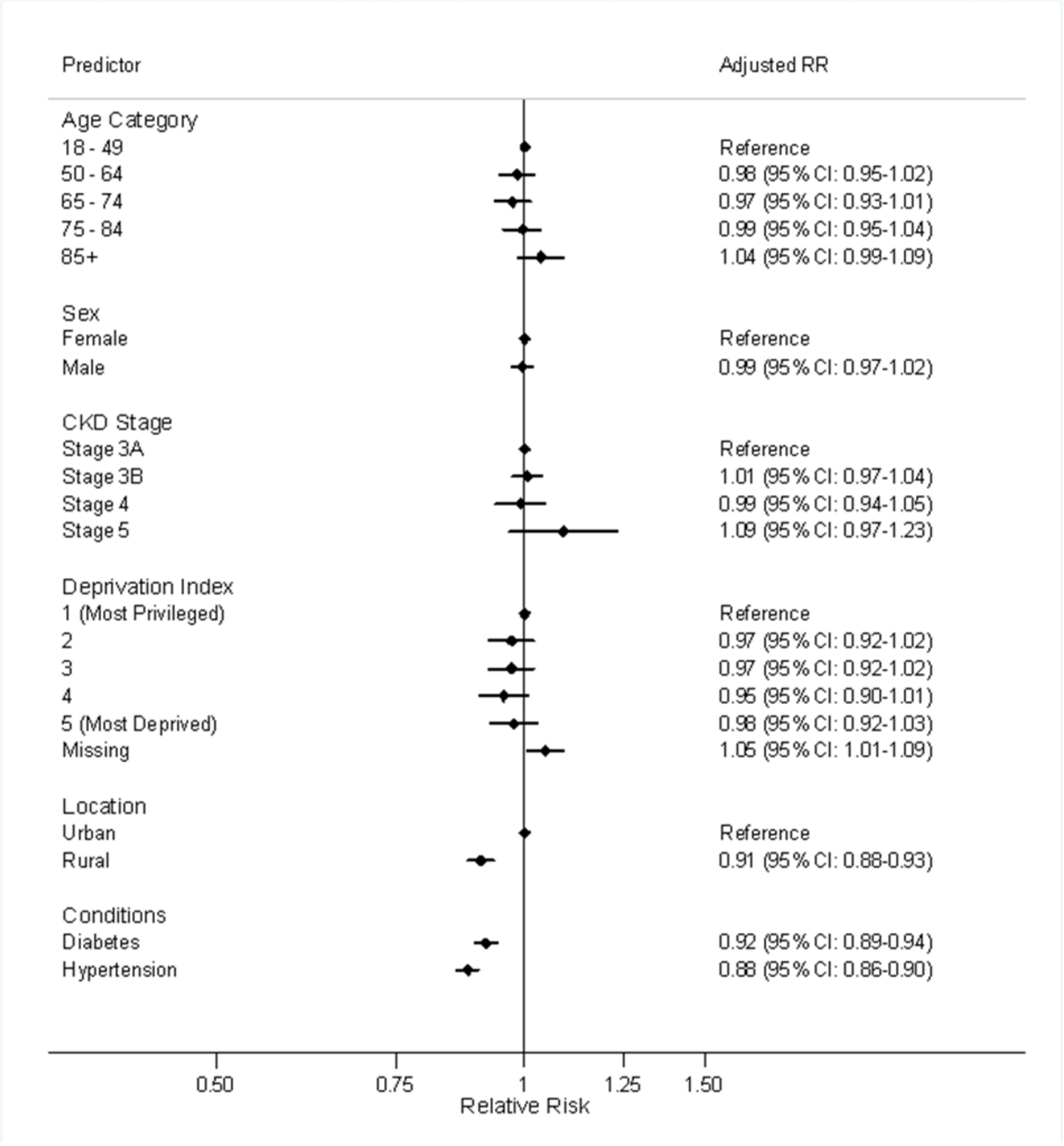

CKD=chronic kidney disease; CI= confidence interval; RR= risk ratio

© 2019 Bello AK et al. *JAMA Network Open.*

eFigure 8. Associations for not achieving a target BP of ≤140/90mmHg, among those with blood pressure measure after CKD diagnosis

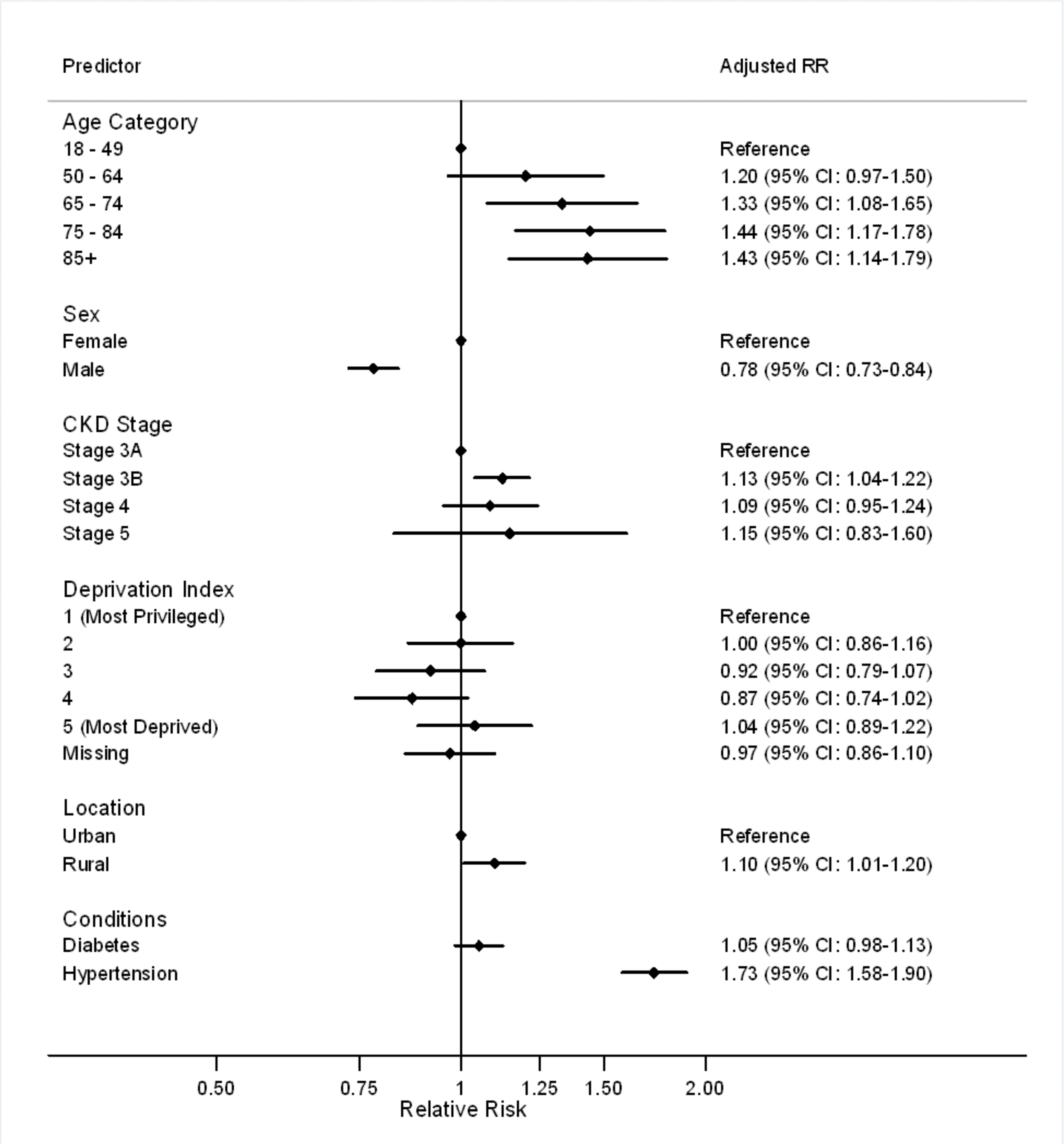

BP= blood pressure; CI= confidence interval; CKD=chronic kidney disease; eGFR=estimated glomerular filtration rate, RR= risk ratio.

© 2019 Bello AK et al. *JAMA Network Open.*

eFigure 9. Associations for not achieving a target BP of ≤130/80mmHg, among those with confirmed CKD and evidence of albuminuria and/or diabetes

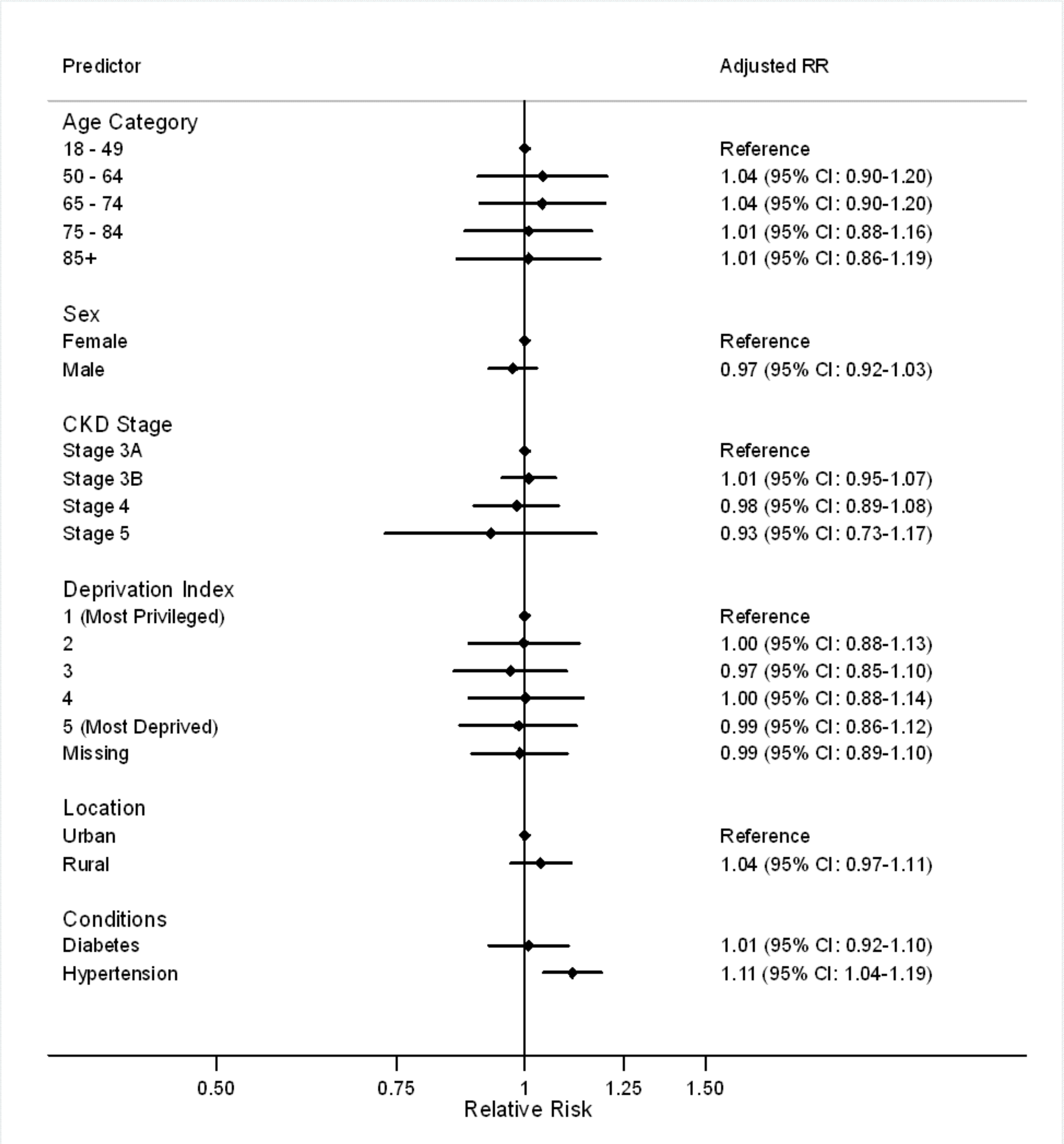

BP= blood pressure; CI= confidence interval; CKD=chronic kidney disease; eGFR=estimated glomerular filtration rate, RR= risk ratio.

© 2019 Bello AK et al. *JAMA Network Open.*

eTable 1. Quality of care indicators for CKD, overall and by comorbid status, and disease stage

Patients receiving UACR test within 6 mos. of initial eGFR < 60 ml/min/1.73 m^2	Overall	CKD without diabetes or hypertension	CKD with diabetes	CKD with hypertension	CKD with diabetes and hypertension	*P*-value
Stage 3A	**n=24,368** 4,134 (17.0)	**n=5,270** 313 (5.9)	**n=2,300** 764 (33.2)	**n=10,749** 936 (8.7)	**n=6,049** 2,121 (35.1)	*p<0.001*
Stage 3B	**n=8,457** 1,757 (20.8)	**n=1,296** 110 (8.5)	**n=841** 270 (32.1)	**n=3,846** 478 (12.4)	**n=2,474** 899 (36.3)	*p<0.001*
Stage 4	**n=2,290** 569 (24.9)	**n=304** 44 (14.5)	**n=266** 80 (30.1)	**n=942** 173 (18.4)	**n=778** 272 (35.0)	*p<0.001*
Stage 5	**n=402** 69 (17.2)	**n=81** 5 (6.2)	**n=77** 15 (19.5)	**n=129** 20 (15.5)	**n=115** 29 (25.2)	*p*=0.005
**Patients receiving UACR test within 6 mos. of initial positive UACR test**	**Overall**	**CKD without diabetes or hypertension**	**CKD with diabetes**	**CKD with hypertension**	**CKD with diabetes and hypertension**	***P*-value**
Stage 3A	**n=1,193** 495 (41.5)	**n=45** 19 (42.2)	**n=240** 93 (38.8)	**n=126** 49 (38.9)	**n=782** 334 (42.7)	*p*=0.663
Stage 3B	**n=221** 96 (43.4)	**n=7** 3 (42.9)	**n=49** 17 (34.7)	**n=27** 16 (59.3)	**n=138** 60 (43.5)	*p*=0.223
Stage 4	**n=51** 25 (49.0)	**n=3** 2 (66.7)	**n=12** 6 (50.0)	**n=10** 3 (30.0)	**n=26** 14 (53.9)	*p*=0.558
Stage 5	**n=17** 7 (41.2)	**n=2** 1 (50.0)	**n=1** 0 (0.0)	**n=2** 1 (50.0)	**n=12** 5 (41.7)	*p*=0.842
**Patients with an outpatient SCr test in the 18 mos. following the confirmation of CKD**	**Overall**	**CKD without diabetes or hypertension**	**CKD with diabetes**	**CKD with hypertension**	**CKD with diabetes and hypertension**	***P*-value**
Stage 3A	**n=21,637** 18,181 (84.0)	**n=4,391** 3,337 (76.0)	**n=2,153** 1,884 (87.5)	**n=9,407** 7,810 (83.0)	**n=5,686** 5,150 (90.6)	*p<0.001*
Stage 3B	**n=7,706** 6,810 (88.4)	**n=1,151** 937 (81.4)	**n=794** 734 (92.4)	**n=3,431** 2,990 (87.2)	**n=2,330** 2,149 (92.2)	*p<0.001*
Stage 4	**n=2,130** 1,922 (90.2)	**n=278** 223 (80.2)	**n=254** 239 (94.1)	**n=857** 768 (89.6)	**n=741** 692 (93.4)	*p<0.001*
Stage 5	**n=357** 301 (84.3)	**n=69** 54 (78.3)	**n=64** 56 (87.5)	**n=117** 98 (83.8)	**n=107** 93 (86.9)	*p*=0.395
**Patients with a UACR test in the 18 mos. following the confirmation of CKD**	**Overall**	**CKD without diabetes or hypertension**	**CKD with diabetes**	**CKD with hypertension**	**CKD with diabetes and hypertension**	***P*-value**
Stage 3A	**n=21,637** 5,497 (25.4)	**n=4,391** 402 (9.2)	**n=2,153** 998 (46.4)	**n=9,407** 1,331 (14.2)	**n=5,686** 2,766 (48.7)	*p<0.001*
Stage 3B	**n=7,706** 2,321 (30.1)	**n=1,151** 131 (11.4)	**n=794** 371 (46.7)	**n=3,431** 657 (19.2)	**n=2,330** 1,162 (49.9)	*p<0.001*
Stage 4	**n=2,130** 708 (33.2)	**n=278** 38 (13.7)	**n=254** 104 (40.9)	**n=857** 212 (24.7)	**n=741** 354 (47.8)	*p<0.001*

© 2019 Bello AK et al. *JAMA Network Open.*

Stage 5	**n=357**<br>67 (18.8)	**n=69**<br>9 (13.0)	**n=64**<br>12 (18.8)	**n=117**<br>18 (15.4)	**n=107**<br>28 (26.2)	*p*=0.102
Patients prescribed an ACEIs or ARBs any time in the 1 year following the confirmation of CKD who have evidence of albuminuria and/or diabetes	Overall	CKD without diabetes or hypertension	CKD with diabetes	CKD with hypertension	CKD with diabetes and hypertension	*P*-value
Stage 3A	**n=8985**<br>4542 (50.6)	**n=123**<br>33 (26.8)	**n=2300**<br>865 (37.6)	**n=518**<br>292 (56.4)	**n=6044**<br>3352 (55.5)	*p<0.001*
Stage 3B	**n=3695**<br>1857 (50.3)	**n=53**<br>19 (35.9)	**n=840**<br>326 (38.8)	**n=330**<br>191 (57.9)	**n=2472**<br>1321 (53.4)	*p<0.001*
Stage 4	**n=1209**<br>512 (42.4)	**n=27**<br>5 (18.5)	**n=266**<br>78 (29.3)	**n=138**<br>59 (42.8)	**n=778**<br>370 (47.6)	*p<0.001*
Stage 5	**n=212**<br>50 (23.6)	**n=5**<br>0 (.0)	**n=77**<br>9 (11.7)	**n=15**<br>8 (53.3)	**n=115**<br>33 (28.7)	*p*=0.001
Patients prescribed a statin any time in the 1 year following the confirmation of CKD	Overall	CKD without diabetes or hypertension	CKD with diabetes	CKD with hypertension	CKD with diabetes and hypertension	*P*-value
Stage 3A	**n=21637**<br>7915 (36.6)	**n=4391**<br>906 (20.6)	**n=2153**<br>943 (43.8)	**n=9407**<br>3184 (33.9)	**n=5686**<br>2882 (50.7)	*p<0.001*
Stage 3B	**n=7706**<br>2894 (37.6)	**n=1151**<br>233 (20.2)	**n=794**<br>334 (42.1)	**n=3431**<br>1143 (33.3)	**n=2330**<br>1184 (50.8)	*p<0.001*
Stage 4	**n=2130**<br>767 (36.0)	**n=278**<br>48 (17.3)	**n=254**<br>102 (40.2)	**n=857**<br>262 (30.6)	**n=741**<br>355 (47.9)	*p<0.001*
Stage 5	**n=357**<br>95 (26.6)	**n=69**<br>11 (15.9)	**n=64**<br>20 (31.3)	**n=117**<br>24 (20.5)	**n=107**<br>40 (37.4)	*p*=0.004
Patients with confirmed CKD who receive an outpatient SCr test 7 to 30 days after initial ACEIs/ARBs prescription date	Overall	CKD without diabetes or hypertension	CKD with diabetes	CKD with hypertension	CKD with diabetes and hypertension	*P*-value
Stage 3A	**n=1605**<br>419 (26.1)	**n=186**<br>53 (28.5)	**n=157**<br>43 (27.4)	**n=793**<br>223 (28.1)	**n=469**<br>100 (21.3)	*p*=0.048
Stage 3B	**n=676**<br>178 (26.3)	**n=62**<br>12 (19.4)	**n=84**<br>20 (23.8)	**n=312**<br>90 (28.9)	**n=218**<br>56 (25.7)	*p*=0.408
Stage 4	**n=157**<br>51 (32.5)	**n=12**<br>4 (33.3)	**n=16**<br>7 (43.8)	**n=57**<br>19 (33.3)	**n=72**<br>21 (29.2)	*p*=0.727
Stage 5	**n=29**<br>11 (37.9)	**n=2**<br>0 (0.0)	**n=6**<br>3 (50.0)	**n=10**<br>2 (20.0)	**n=11**<br>6 (54.6)	*p*=0.236

ACEI=angiotensin-converting enzyme inhibitors; ARB=angiotensin-receptor blockers; CKD=chronic kidney disease; eGFR=estimated glomerular filtration rate; SCr=serum creatinine; UACR=urine albumin-to-creatinine ratio.

© 2019 Bello AK et al. *JAMA Network Open.*

eTable 2. Quality of care indicators for CKD, overall and by comorbid status and age categories

Patients receiving UACR test within 6 mos. of initial eGFR < 60 ml/min/1.73 m^2	Overall	CKD without diabetes or hypertension	CKD with diabetes	CKD with hypertension	CKD with diabetes and hypertension	*P*-value
18 – 49	**n=1,698**<br>314 (18.5)	**n=688**<br>55 (8.0)	**n=295**<br>71 (24.1)	**n=434**<br>85 (19.6)	**n=281**<br>103 (36.7)	*p*<0.001
50 – 64	**n=6,611**<br>1,497 (22.6)	**n=1,532**<br>139 (9.1)	**n=795**<br>283 (35.6)	**n=2,347**<br>304 (13.0)	**n=1,937**<br>771 (39.8)	*p*<0.001
65 – 74	**n=10,052**<br>2,121 (21.1)	**n=1,697**<br>118 (7.0)	**n=1,027**<br>363 (35.4)	**n=4,198**<br>464 (11.1)	**n=3,130**<br>1,176 (37.6)	*p*<0.001
75 – 84	**n=11,919**<br>1,983 (16.6)	**n=1,927**<br>117 (6.1)	**n=1,034**<br>325 (31.4)	**n=5,867**<br>534 (9.1)	**n=3,091**<br>1,007 (32.6)	*p*<0.001
85+	**n=5,237**<br>614 (11.7)	**n=1,107**<br>43 (3.9)	**n=333**<br>87 (26.1)	**n=2,820**<br>220 (7.8)	**n=977**<br>264 (27.0)	*p*<0.001
Patients receiving UACR test within 6 mos. of initial positive UACR test	Overall	CKD without diabetes or hypertension	CKD with diabetes	CKD with hypertension	CKD with diabetes and hypertension	*P*-value
18 – 49	**n=2,377**<br>975 (41.0)	**n=286**<br>145 (50.7)	**n=970**<br>380 (39.2)	**n=281**<br>115 (40.9)	**n=840**<br>335 (39.9)	*p*=0.005
50 – 64	**n=4,028**<br>1,596 (39.6)	**n=153**<br>67 (43.8)	**n=1,208**<br>460 (38.1)	**n=418**<br>155 (37.1)	**n=2,249**<br>914 (40.6)	*p*=0.220
65 – 74	**n=2,291**<br>866 (37.8)	**n=85**<br>24 (28.2)	**n=502**<br>177 (35.3)	**n=273**<br>81 (29.7)	**n=1,431**<br>584 (40.8)	*p*<0.001
75 – 84	**n=1,141**<br>442 (38.7)	**n=48**<br>16 (33.3)	**n=225**<br>101 (44.9)	**n=158**<br>48 (30.4)	**n=710**<br>277 (39.0)	*p*=0.031
85+	**n=191**<br>75 (39.3)	**n=6**<br>2 (33.3)	**n=37**<br>12 (32.4)	**n=32**<br>14 (43.8)	**n=116**<br>47 (40.5)	*p*=0.763
Patients with an outpatient SCr test in the 18 mos. following the confirmation of CKD	Overall	CKD without diabetes or hypertension	CKD with diabetes	CKD with hypertension	CKD with diabetes and hypertension	*P*-value
18 – 49	**n=1,483**<br>1,223 (82.5)	**n=577**<br>445 (77.1)	**n=276**<br>245 (88.8)	**n=365**<br>300 (82.2)	**n=265**<br>233 (87.9)	*p*<0.001
50 – 64	**n=5,904**<br>4,923 (83.4)	**n=1,257**<br>938 (74.6)	**n=741**<br>657 (88.7)	**n=2,093**<br>1,703 (81.4)	**n=1,813**<br>1,625 (89.6)	*p*<0.001
65 – 74	**n=9,067**	**n=1,442**	**n=968**	**n=3,693**	**n=2,964**	

© 2019 Bello AK et al. *JAMA Network Open.*

	7887 (87.0)	1132 (78.5)	875 (90.4)	3132 (84.8)	2748 (92.7)	*p*<0.001
75 – 84	**n=10,715** 9,276 (86.6)	**n=1,658** 1,307 (78.8)	**n=965** 859 (89.0)	**n=5,175** 4,453 (86.1)	**n=2,917** 2,657 (91.1)	*p*<0.001
85+	**n=4,668** 3,912 (83.8)	**n=956** 730 (76.4)	**n=315** 277 (87.9)	**n=2,488** 2,080 (83.6)	**n=909** 825 (90.8)	*p*<0.001
Patients with a UACR test in the 18 mos. following the confirmation of CKD	Overall	CKD without diabetes or hypertension	CKD with diabetes	CKD with hypertension	CKD with diabetes and hypertension	*P*-value
18 – 49	**n=1,483** 372 (25.1)	**n=577** 72 (12.5)	**n=276** 92 (33.3)	**n=365** 89 (24.4)	**n=265** 119 (44.9)	*p*<0.001
50 – 64	**n=5,904** 1,940 (32.9)	**n=1,257** 153 (12.2)	**n=741** 379 (51.2)	**n=2,093** 425 (20.3)	**n=1,813** 983 (54.2)	*p*<0.001
65 – 74	**n=9,067** 2,847 (31.4)	**n=1,442** 161 (11.2)	**n=968** 482 (49.8)	**n=3,693** 654 (17.7)	**n=2,964** 1,550 (52.3)	*p*<0.001
75 – 84	**n=10,715** 2,642 (24.7)	**n=1,658** 145 (8.8)	**n=965** 426 (44.2)	**n=5,175** 754 (14.6)	**n=2,917** 1,317 (45.2)	*p*<0.001
85+	**n=4,668** 798 (17.1)	**n=956** 50 (5.2)	**n=315** 106 (33.7)	**n=2,488** 297 (11.9)	**n=909** 345 (38.0)	*p*<0.001
Patients prescribed a Statin any time in the 1 year following the confirmation of CKD	Overall	CKD without diabetes or hypertension	CKD with diabetes	CKD with hypertension	CKD with diabetes and hypertension	*P*-value
18 – 49	**n=1,483** 259 (17.5)	**n=577** 36 (6.2)	**n=276** 58 (21.0)	**n=365** 60 (16.4)	**n=265** 105 (39.6)	*p<0.001*
50 – 64	**n=5,904** 2,094 (35.5)	**n=1,257** 235 (18.7)	**n=741** 315 (42.5)	**n=2,093** 652 (31.2)	**n=1,813** 892 (49.2)	*p<0.001*
65 – 74	**n=9,067** 3,792 (41.8)	**n=1,442** 361 (25.0)	**n=968** 463 (47.8)	**n=3,693** 1,391 (37.7)	**n=2,964** 1,577 (53.2)	*p<0.001*
75 – 84	**n=10,715** 4,267 (39.8)	**n=1,658** 420 (25.3)	**n=965** 472 (48.9)	**n=5,175** 1,851 (35.8)	**n=2,917** 1,524 (52.3)	*p<0.001*
85+	**n=4,668** 1,260 (27.0)	**n=956** 146 (15.3)	**n=315** 91 (28.9)	**n=2,488** 659 (26.5)	**n=909** 364 (40.0)	*p<0.001*
Patients prescribed an ACEI or ARB any time in the 1 year following the confirmation of CKD who have evidence of proteinuria and/or diabetes	Overall	CKD without diabetes or hypertension	CKD with diabetes	CKD with hypertension	CKD with diabetes and hypertension	*P*-value

© 2019 Bello AK et al. *JAMA Network Open.*

18 – 49	**n=2,881**<br>222 (7.7)	**n=43**<br>2 (4.7)	**n=1,471**<br>53 (3.6)	**n=84**<br>42 (50.0)	**n=1,283**<br>125 (9.7)	*p<0.001*
50 – 64	**n=6,822**<br>1,461 (21.4)	**n=37**<br>8 (21.6)	**n=2,172**<br>309 (14.2)	**n=202**<br>109 (54.0)	**n=4,411**<br>1,035 (23.5)	*p<0.001*
65 – 74	**n=6,327**<br>2,327 (36.8)	**n=54**<br>19 (35.2)	**n=1,546**<br>409 (26.5)	**n=275**<br>149 (54.2)	**n=4,452**<br>1,750 (39.3)	*p<0.001*
75 – 84	**n=5,262**<br>2,328 (44.2)	**n=60**<br>23 (38.3)	**n=1219**<br>415 (34.0)	**n=328**<br>194 (59.2)	**n=3,655**<br>1,696 (46.4)	*p<0.001*
85+	**n=1,541**<br>626 (40.6)	**n=15**<br>5 (33.3)	**n=361**<br>92 (25.5)	**n=114**<br>57 (50.0)	**n=1,051**<br>472 (44.9)	*p<0.001*
Patients who receive an outpatient SCr test 7 to 30 days after incident ACEI/ARB prescription date in patients with confirmed CKD	Overall	CKD without diabetes or hypertension	CKD with diabetes	CKD with hypertension	CKD with diabetes and hypertension	*P*-value
18 – 49	**n=91**<br>29 (31.9)	**n=13**<br>3 (23.1)	**n=13**<br>2 (15.4)	**n=36**<br>13 (36.1)	**n=29**<br>11 (37.9)	*p*=0.411
50 – 64	**n=429**<br>101 (23.5)	**n=42**<br>13 (31.0)	**n=55**<br>15 (27.3)	**n=199**<br>51 (25.6)	**n=133**<br>22 (16.5)	*p*=0.121
65 – 74	**n=713**<br>199 (27.9)	**n=71**<br>18 (25.4)	**n=77**<br>23 (29.9)	**n=313**<br>85 (27.2)	**n=252**<br>73 (29.0)	*p*=0.895
75 – 84	**n=896**<br>234 (26.1)	**n=99**<br>26 (26.3)	**n=96**<br>25 (26.0)	**n=426**<br>119 (27.9)	**n=275**<br>64 (23.3)	*p*=0.597
85+	**n=338**<br>96 (28.4)	**n=37**<br>9 (24.3)	**n=22**<br>8 (36.4)	**n=198**<br>66 (33.3)	**n=81**<br>13 (16.1)	*p*=0.024

ACEI=angiotensin-converting enzyme inhibitors; ARBs=angiotensin-receptor blockers; CKD=chronic kidney disease; eGFR=estimated glomerular filtration rate; SCr=serum creatinine; UACR=urine albumin-to-creatinine ratio.

© 2019 Bello AK et al. *JAMA Network Open.*

eTable 3. Quality of care indicators for CKD, overall and by comorbid status, and sex

Patients receiving UACR test within 6 mos. of initial eGFR < 60 ml/min/1.73 m^2	Overall	CKD without diabetes or hypertension	CKD with diabetes	CKD with hypertension	CKD with diabetes and hypertension	*P*-value
Female	**n=21,246**<br>3,292 (15.5)	**n=4,273**<br>235 (5.5)	**n=1,720**<br>490 (28.5)	**n=10,045**<br>883 (8.8)	**n=5,208**<br>1,684 (32.3)	*p*<0.001
Male	**n=14,271**<br>3,237 (22.7)	**n=2,678**<br>237 (8.9)	**n=1,764**<br>639 (36.2)	**n=5,621**<br>724 (12.9)	**n=4,208**<br>1,637 (38.9)	*p*<0.001
Patients receiving UACR test within 6 mos. of initial positive UACR test	Overall	CKD without diabetes or hypertension	CKD with diabetes	CKD with hypertension	CKD with diabetes and hypertension	*P*-value
Female	**n=4,439**<br>1,730 (39.0)	**n=339**<br>144 (42.5)	**n=1,232**<br>465 (37.7)	**n=619**<br>205 (33.1)	**n=2,249**<br>916 (40.7)	*p*=0.002
Male	**n=5,589**<br>2,224 (39.8)	**n=239**<br>110 (46.0)	**n=1,710**<br>665 (38.9)	**n=543**<br>208 (38.3)	**n=3,097**<br>1,241 (40.1)	*p=0.168*
Patients with an outpatient SCr test in the 18 mos. following the confirmation of CKD	Overall	CKD without diabetes or hypertension	CKD with diabetes	CKD with hypertension	CKD with diabetes and hypertension	*P*-value
Female	**n=18,916**<br>16,069 (85.0)	**n=3,556**<br>2,748 (77.3)	**n=1,604**<br>1,424 (88.8)	**n=8,847**<br>7,420 (83.9)	**n=4,909**<br>4,477 (91.2)	*p*<0.001
Male	**n=12,921**<br>11,152 (86.3)	**n=2,334**<br>1,804 (77.3)	**n=1,661**<br>1,489 (89.6)	**n=4,967**<br>4,248 (85.5)	**n=3,959**<br>3,611 (91.2)	*p*<0.001
Patients with a UACR test in the 18 mos. following the confirmation of CKD	Overall	CKD without diabetes or hypertension	CKD with diabetes	CKD with hypertension	CKD with diabetes and hypertension	*P*-value
Female	**n=18,916**<br>4,485 (23.7)	**n=3,556**<br>299 (8.4)	**n=1,604**<br>652 (40.7)	**n=8,847**<br>1,272 (14.4)	**n=4,909**<br>2,262 (46.1)	*p*<0.001
Male	**n=12,921**<br>4,114 (31.8)	**n=2,334**<br>282 (12.1)	**n=1,661**<br>833 (50.2)	**n=4,967**<br>947 (19.1)	**n=3,959**<br>2,052 (51.8)	*p*<0.001
Patients prescribed an ACEIs or ARBs any time in the 1 year following the confirmation of CKD who have evidence of proteinuria and/or diabetes	Overall	CKD without diabetes or hypertension	CKD with diabetes	CKD with hypertension	CKD with diabetes and hypertension	*P*-value
Female	**n=11,115**	**n=100**	**n=3,070**	**n=525**	**n=7,420**	

© 2019 Bello AK et al. *JAMA Network Open.*

	3,710 (33.4)	20 (20.0)	583 (19.0)	288 (54.9)	2,819 (38.0)	*p*<0.001
Male	**n=11,718** 3,254 (27.8)	**n=109** 37 (33.9)	**n=3,699** 695 (18.8)	**n=478** 263 (55.0)	**n=7,432** 2,259 (30.4)	*p*<0.001
Patients prescribed a statin any time in the 1 year following the confirmation of CKD	Overall	CKD without diabetes or hypertension	CKD with diabetes	CKD with hypertension	CKD with diabetes and hypertension	*P*-value
Female	**n=18,916** 6,297 (33.3)	**n=3,556** 600 (16.9)	**n=1,604** 619 (38.6)	**n=8,847** 2,685 (30.4)	**n=4,909** 2,393 (48.8)	*p*<0.001
Male	**n=12,921** 5,375 (41.6)	**n=2,334** 598 (25.6)	**n=1,661** 780 (47.0)	**n=4,967** 1,928 (38.8)	**n=3,959** 2,069 (52.3)	*p*<0.001
Patients with confirmed CKD who receive an outpatient SCr test 7 to 30 days after initial ACEIs/ARBs prescription date	Overall	CKD without diabetes or hypertension	CKD with diabetes	CKD with hypertension	CKD with diabetes and hypertension	*P*-value
Female	**n=1465** 396 (27.0)	**n=135** 32 (23.7)	**n=113** 35 (31.0)	**n=766** 221 (28.9)	**n=451** 108 (24.0)	*p*=0.164
Male	**n=1002** 263 (26.3)	**n=127** 37 (29.1)	**n=150** 38 (25.3)	**n=406** 113 (27.8)	**n=319** 75 (23.5)	*p*=0.499

ACEI=angiotensin-converting enzyme inhibitors; ARBs=angiotensin-receptor blockers; CKD=chronic kidney disease; eGFR=estimated glomerular filtration rate; SCr=serum creatinine; UACR=urine albumin-to-creatinine ratio.

© 2019 Bello AK et al. *JAMA Network Open.*

eTable 4. Variations of quality of care indicators for CKD, across physician characteristics (age and gender)

Age <40 women	Overall	CKD without diabetes or hypertension	CKD with diabetes	CKD with hypertension	CKD with diabetes and hypertension
Patients receiving UACR test within 6 mos. of initial eGFR < 60 ml/min/1.73 m^2	0.443	0.521	0.415	0.381	0.464
Patients with an outpatient SCr test in the 18 mos. following the confirmation of CKD	0.855	0.761	0.890	0.852	0.908
Patients with a UACR test in the 18 mos. following the confirmation of CKD	0.338	0.151	0.546	0.228	0.568
Patients prescribed a statin any time in the 1 year following the confirmation of CKD	0.413	0.218	0.475	0.386	0.558
Patients prescribed an ACEIs or ARBs any time in the 1 year following the confirmation of CKD who have evidence of proteinuria and/or diabetes	0.319	0.229	0.174	0.607	0.355
Patients with confirmed CKD who receive an outpatient SCr test 7 to 30 days after initial ACEIs/ARBs prescription date	0.347	0.382	0.326	0.365	0.316
Patients receiving BP measurement at any time	0.7594	0.6904	0.751	0.781	0.7726
Patients receiving BP measurement within 6 mos. of initial eGFR < 60 ml/min/1.73 m^2	0.287	0.265	0.145	0.360	0.277
Patients with eGFR < 60 ml/min/1.73 m^2 achieving a target BP of ≤ 140/90mmHg	0.8199	0.9048	0.844	0.7979	0.8028
Patients with eGFR < 60 ml/min/1.73 m^2 achieving a target BP of ≤ 130/80mmHg, who have evidence of proteinuria and/or diabetes	0.5849	0.8182	0.6223	0.4593	0.5889
Patients with eGFR <60 ml/min/1.73 m^2 and diabetes who have HbA1c tested within the 1st and 2nd years					
0 - 1 year	0.877		0.8966		0.8714
1 - 2 year	0.6727		0.6749		0.6721
**Age 40-49 women**	**Overall**	**CKD without diabetes or hypertension**	**CKD with diabetes**	**CKD with hypertension**	**CKD with diabetes and hypertension**
Patients receiving UACR test within 6 mos. of initial eGFR < 60 ml/min/1.73 m^2	0.418	0.584	0.359	0.426	0.427
Patients with an outpatient SCr test in the 18 mos. following the confirmation of CKD	0.862	0.797	0.913	0.846	0.916

© 2019 Bello AK et al. *JAMA Network Open.*

Patients with a UACR test in the 18 mos. following the confirmation of CKD	0.267	0.110	0.467	0.154	0.486
Patients prescribed a statin any time in the 1 year following the confirmation of CKD	0.398	0.226	0.440	0.368	0.551
Patients prescribed an ACEIs or ARBs any time in the 1 year following the confirmation of CKD who have evidence of proteinuria and/or diabetes	0.342	0.185	0.181	0.634	0.401
Patients with confirmed CKD who receive an outpatient SCr test 7 to 30 days after initial ACEIs/ARBs prescription date	0.232	0.097	0.333	0.281	0.180
Patients receiving BP measurement at any time	0.742	0.6437	0.7684	0.7668	0.7563
Patients receiving BP measurement within 6 mos. of initial eGFR < 60 ml/min/1.73 m^2	0.292	0.231	0.173	0.369	0.293
Patients with eGFR < 60 ml/min/1.73 m^2 achieving a target BP of ≤ 140/90mmHg	0.8073	0.8902	0.8295	0.7856	0.7854
Patients with eGFR < 60 ml/min/1.73 m^2 achieving a target BP of ≤ 130/80mmHg, who have evidence of proteinuria and/or diabetes	0.6055	0.75	0.6432	0.6806	0.5789
Patients with eGFR <60 ml/min/1.73 m^2 and diabetes who have HbA1c tested within the 1st and 2nd years					
0 - 1 year	0.8393		0.8709		0.8282
1 - 2 year	0.6643		0.6868		0.6564
Age 50-59 women	Overall	CKD without diabetes or hypertension	CKD with diabetes	CKD with hypertension	CKD with diabetes and hypertension
Patients receiving UACR test within 6 mos. of initial eGFR < 60 ml/min/1.73 m^2	0.427	0.479	0.428	0.418	0.424
Patients with an outpatient SCr test in the 18 mos. following the confirmation of CKD	0.865	0.781	0.907	0.855	0.924
Patients with a UACR test in the 18 mos. following the confirmation of CKD	0.237	0.058	0.426	0.124	0.464
Patients prescribed a statin any time in the 1 year following the confirmation of CKD	0.387	0.198	0.469	0.343	0.557
Patients prescribed an ACEIs or ARBs any time in the 1 year following the confirmation of CKD who have evidence of proteinuria and/or diabetes	0.330	0.375	0.209	0.589	0.376
Patients with confirmed CKD who receive an outpatient SCr test 7 to 30 days after initial ACEIs/ARBs prescription date	0.272	0.321	0.194	0.284	0.265

© 2019 Bello AK et al. *JAMA Network Open.*

Patients receiving BP measurement at any time	0.8109	0.6854	0.8123	0.833	0.8528
Patients receiving BP measurement within 6 mos. of initial eGFR < 60 ml/min/1.73 m^2	0.346	0.267	0.231	0.432	0.345
Patients with eGFR < 60 ml/min/1.73 m^2 achieving a target BP of ≤ 140/90mmHg	0.801	0.8571	0.8784	0.7697	0.7858
Patients with eGFR < 60 ml/min/1.73 m^2 achieving a target BP of ≤ 130/80mmHg, who have evidence of proteinuria and/or diabetes	0.6294	0.5385	0.7212	0.6182	0.5949
Patients with eGFR <60 ml/min/1.73 m^2 and diabetes who have HbA1c tested within the 1st and 2nd years					
0 - 1 year	0.893		0.9056		0.8877
1 - 2 year	0.7051		0.6931		0.7101
Age 60+ women	Overall	CKD without diabetes or hypertension	CKD with diabetes	CKD with hypertension	CKD with diabetes and hypertension
Patients receiving UACR test within 6 mos. of initial eGFR < 60 ml/min/1.73 m^2	0.422	0.250	0.471	0.417	0.447
Patients with an outpatient SCr test in the 18 mos. following the confirmation of CKD	0.831	0.722	0.833	0.843	0.906
Patients with a UACR test in the 18 mos. following the confirmation of CKD	0.260	0.124	0.417	0.184	0.509
Patients prescribed a statin any time in the 1 year following the confirmation of CKD	0.362	0.227	0.292	0.377	0.472
Patients prescribed an ACEIs or ARBs any time in the 1 year following the confirmation of CKD who have evidence of proteinuria and/or diabetes	0.298	0.667	0.154	0.684	0.294
Patients with confirmed CKD who receive an outpatient SCr test 7 to 30 days after initial ACEIs/ARBs prescription date	0.500	0.000	0.667	0.500	0.800
Patients receiving BP measurement at any time	0.8472	0.8056	0.6923	0.866	0.8853
Patients receiving BP measurement within 6 mos. of initial eGFR < 60 ml/min/1.73 m^2	0.333	0.306	0.173	0.399	0.298
Patients with eGFR < 60 ml/min/1.73 m^2 achieving a target BP of ≤ 140/90mmHg	0.851	0.9074	1	0.8075	0.8846
Patients with eGFR < 60 ml/min/1.73 m^2 achieving a target BP of ≤ 130/80mmHg, who have evidence of proteinuria and/or diabetes	0.6923	0	0.625	0.6923	0.7206
Patients with eGFR <60 ml/min/1.73 m^2 and diabetes who have HbA1c tested within the 1st and 2nd years					

© 2019 Bello AK et al. *JAMA Network Open.*

0 - 1 year	0.8865		0.7778		0.9123
1 - 2 year	0.6525		0.5556		0.6754
Age <40 men	Overall	CKD without diabetes or hypertension	CKD with diabetes	CKD with hypertension	CKD with diabetes and hypertension
Patients receiving UACR test within 6 mos. of initial eGFR < 60 ml/min/1.73 m^2	0.387	0.326	0.370	0.336	0.406
Patients with an outpatient SCr test in the 18 mos. following the confirmation of CKD	0.856	0.788	0.874	0.849	0.903
Patients with a UACR test in the 18 mos. following the confirmation of CKD	0.286	0.096	0.456	0.192	0.487
Patients prescribed a statin any time in the 1 year following the confirmation of CKD	0.356	0.211	0.453	0.323	0.463
Patients prescribed an ACEIs or ARBs any time in the 1 year following the confirmation of CKD who have evidence of proteinuria and/or diabetes	0.291	0.333	0.200	0.457	0.315
Patients with confirmed CKD who receive an outpatient SCr test 7 to 30 days after initial ACEIs/ARBs prescription date	0.312	0.286	0.425	0.299	0.296
Patients receiving BP measurement at any time	0.7355	0.677	0.7169	0.7337	0.7711
Patients receiving BP measurement within 6 mos. of initial eGFR < 60 ml/min/1.73 m^2	0.287	0.254	0.183	0.356	0.271
Patients with eGFR < 60 ml/min/1.73 m^2 achieving a target BP of ≤ 140/90mmHg	0.8279	0.8917	0.8685	0.8041	0.8159
Patients with eGFR < 60 ml/min/1.73 m^2 achieving a target BP of ≤ 130/80mmHg, who have evidence of proteinuria and/or diabetes	0.5994	0.6	0.7041	0.5393	0.5753
Patients with eGFR <60 ml/min/1.73 m^2 and diabetes who have HbA1c tested within the 1st and 2nd years					
0 - 1 year	0.8913		0.8747		0.8972
1 - 2 year	0.7217		0.6867		0.734
Age 40-49 men	Overall	CKD without diabetes or hypertension	CKD with diabetes	CKD with hypertension	CKD with diabetes and hypertension
Patients receiving UACR test within 6 mos. of initial eGFR < 60 ml/min/1.73 m^2	0.375	0.480	0.335	0.341	0.395
Patients with an outpatient SCr test in the 18 mos. following the confirmation of CKD	0.860	0.782	0.888	0.853	0.910

© 2019 Bello AK et al. *JAMA Network Open.*

Patients with a UACR test in the 18 mos. following the confirmation of CKD	0.235	0.093	0.398	0.121	0.438
Patients prescribed a statin any time in the 1 year following the confirmation of CKD	0.345	0.225	0.357	0.305	0.481
Patients prescribed an ACEIs or ARBs any time in the 1 year following the confirmation of CKD who have evidence of proteinuria and/or diabetes	0.280	0.067	0.152	0.449	0.334
Patients with confirmed CKD who receive an outpatient SCr test 7 to 30 days after initial ACEIs/ARBs prescription date	0.211	0.194	0.269	0.243	0.156
Patients receiving BP measurement at any time	0.7203	0.5985	0.7067	0.7488	0.7565
Patients receiving BP measurement within 6 mos. of initial eGFR < 60 ml/min/1.73 m^2	0.285	0.216	0.161	0.365	0.291
Patients with eGFR < 60 ml/min/1.73 m^2 achieving a target BP of ≤ 140/90mmHg	0.8234	0.9137	0.8725	0.7927	0.8085
Patients with eGFR < 60 ml/min/1.73 m^2 achieving a target BP of ≤ 130/80mmHg, who have evidence of proteinuria and/or diabetes	0.5882	0.3333	0.6963	0.4615	0.5627
Patients with eGFR <60 ml/min/1.73 m^2 and diabetes who have HbA1c tested within the 1st and 2nd years					
0 - 1 year	0.8459		0.8278		0.8528
1 - 2 year	0.6636		0.6195		0.6803
Age 50-59 men	Overall	CKD without diabetes or hypertension	CKD with diabetes	CKD with hypertension	CKD with diabetes and hypertension
Patients receiving UACR test within 6 mos. of initial eGFR < 60 ml/min/1.73 m^2	0.368	0.229	0.413	0.237	0.381
Patients with an outpatient SCr test in the 18 mos. following the confirmation of CKD	0.857	0.766	0.897	0.847	0.922
Patients with a UACR test in the 18 mos. following the confirmation of CKD	0.275	0.087	0.500	0.159	0.501
Patients prescribed a statin any time in the 1 year following the confirmation of CKD	0.379	0.206	0.456	0.348	0.522
Patients prescribed an ACEIs or ARBs any time in the 1 year following the confirmation of CKD who have evidence of proteinuria and/or diabetes	0.320	0.381	0.218	0.556	0.354
Patients with confirmed CKD who receive an outpatient SCr test 7 to 30 days after initial ACEIs/ARBs prescription date	0.237	0.298	0.175	0.251	0.222

© 2019 Bello AK et al. *JAMA Network Open.*

Patients receiving BP measurement at any time	0.7631	0.6645	0.7715	0.7742	0.7996
Patients receiving BP measurement within 6 mos. of initial eGFR < 60 ml/min/1.73 m^2	0.300	0.255	0.207	0.362	0.295
Patients with eGFR < 60 ml/min/1.73 m^2 achieving a target BP of ≤ 140/90mmHg	0.8059	0.8765	0.8779	0.7763	0.7807
Patients with eGFR < 60 ml/min/1.73 m^2 achieving a target BP of ≤ 130/80mmHg, who have evidence of proteinuria and/or diabetes	0.5976	0.8148	0.6775	0.5411	0.5652
Patients with eGFR <60 ml/min/1.73 m^2 and diabetes who have HbA1c tested within the 1st and 2nd years					
0 - 1 year	0.8446		0.8313		0.8506
1 - 2 year	0.6496		0.63		0.6583
Age 60+ men	Overall	CKD without diabetes or hypertension	CKD with diabetes	CKD with hypertension	CKD with diabetes and hypertension
Patients receiving UACR test within 6 mos. of initial eGFR < 60 ml/min/1.73 m^2	0.318	0.275	0.316	0.250	0.335
Patients with an outpatient SCr test in the 18 mos. following the confirmation of CKD	0.865	0.790	0.891	0.852	0.924
Patients with a UACR test in the 18 mos. following the confirmation of CKD	0.235	0.065	0.412	0.120	0.451
Patients prescribed a statin any time in the 1 year following the confirmation of CKD	0.379	0.195	0.468	0.341	0.522
Patients prescribed an ACEIs or ARBs any time in the 1 year following the confirmation of CKD who have evidence of proteinuria and/or diabetes	0.351	0.300	0.251	0.524	0.393
Patients with confirmed CKD who receive an outpatient SCr test 7 to 30 days after initial ACEIs/ARBs prescription date	0.276	0.280	0.286	0.324	0.186
Patients receiving BP measurement at any time	0.8328	0.6463	0.8667	0.8458	0.8992
Patients receiving BP measurement within 6 mos. of initial eGFR < 60 ml/min/1.73 m^2	0.300	0.255	0.207	0.362	0.295
Patients with eGFR < 60 ml/min/1.73 m^2 achieving a target BP of ≤ 140/90mmHg	0.8155	0.8996	0.8791	0.7891	0.7933
Patients with eGFR < 60 ml/min/1.73 m^2 achieving a target BP of ≤ 130/80mmHg, who have evidence of proteinuria and/or diabetes	0.5895	0.7273	0.6847	0.4762	0.5599
Patients with eGFR <60 ml/min/1.73 m^2 and diabetes who have HbA1c tested within the 1st and 2nd years					

© 2019 Bello AK et al. *JAMA Network Open.*

0 - 1 year	0.9023	0.9189	0.895
1 - 2 year	0.7069	0.6405	0.7358

ACEI=angiotensin-converting enzyme inhibitors; ARBs=angiotensin-receptor blockers; CKD=chronic kidney disease; eGFR=estimated glomerular filtration rate; SCr=serum creatinine; UACR=urine albumin-to-creatinine ratio.

© 2019 Bello AK et al. *JAMA Network Open.*
